# Connections between serum Trimethylamine N-Oxide (TMAO), a gut-derived metabolite, and vascular biomarkers evaluating arterial stiffness and subclinical atherosclerosis in children with obesity

**DOI:** 10.3389/fendo.2023.1253584

**Published:** 2023-10-02

**Authors:** Monica Simina Mihuta, Corina Paul, Andreea Borlea, Cristina Mihaela Roi, Denisa Pescari, Oana-Alexandra Velea-Barta, Ioana Mozos, Dana Stoian

**Affiliations:** ^1^ Department of Doctoral Studies, Victor Babes University of Medicine and Pharmacy, Timisoara, Romania; ^2^ Center of Molecular Research in Nephrology and Vascular Disease, Faculty of Medicine, Victor Babes University of Medicine and Pharmacy, Timisoara, Romania; ^3^ Department of Pediatrics, Victor Babes University of Medicine and Pharmacy, Timisoara, Romania; ^4^ 2nd Department of Internal Medicine, Victor Babes University of Medicine and Pharmacy, Timisoara, Romania; ^5^ 3rd Department of Odontotherapy and Endodontics, Faculty of Dental Medicine, Victor Babes University of Medicine and Pharmacy, Timisoara, Romania; ^6^ Department of Functional Sciences—Pathophysiology, Center for Translational Research and Systems Medicine, Victor Babes University of Medicine and Pharmacy, Timisoara, Romania

**Keywords:** arterial stiffness, atherosclerosis, cardiovascular risk, carotid intima-media thickness, childhood obesity, high blood pressure, pulse wave velocity, TMAO

## Abstract

**Introduction:**

Childhood obesity leads to early subclinical atherosclerosis and arterial stiffness. Studying biomarkers like trimethylamine N-oxide (TMAO), linked to cardio-metabolic disorders in adults, is crucial to prevent long-term cardiovascular issues.

**Methods:**

The study involved 70 children aged 4 to 18 (50 obese, 20 normal-weight). Clinical examination included BMI, waist measurements, puberty stage, the presence of acanthosis nigricans, and irregular menstrual cycles. Subclinical atherosclerosis was assessed by measuring the carotid intima-media thickness (CIMT), and the arterial stiffness was evaluated through surrogate markers like the pulse wave velocity (PWV), augmentation index (AIx), and peripheral and central blood pressures. The blood biomarkers included determining the values of TMAO, HOMA-IR, and other usual biomarkers investigating metabolism.

**Results:**

The study detected significantly elevated levels of TMAO in obese children compared to controls. TMAO presented positive correlations to BMI, waist circumference and waist-to-height ratio and was also observed as an independent predictor of all three parameters. Significant correlations were observed between TMAO and vascular markers such as CIMT, PWV, and peripheral BP levels. TMAO independently predicts CIMT, PWV, peripheral BP, and central SBP levels, even after adding BMI, waist circumference, waist-to-height ratio, puberty development and age in the regression model. Obese children with high HOMA-IR presented a greater weight excess and significantly higher vascular markers, but TMAO levels did not differ significantly from the obese with HOMA-IR<cut-offs. TMAO did not correlate to HOMA-IR and insulin levels but presented a negative correlation to fasting glucose levels. An increase in TMAO was shown to be associated with an increase in the probability of the presence of acanthosis nigricans. TMAO levels are not influenced by other blood biomarkers.

**Conclusion:**

Our study provides compelling evidence supporting the link between serum TMAO, obesity, and vascular damage in children. These findings highlight the importance of further research to unravel the underlying mechanisms of this connection.

## 1 Introduction

Childhood obesity, defined by an excess of adipose tissue resulting from an imbalance between energy intake and expenditure, represents an engine of metabolic disruptions (1) associated with the progression of subclinical vascular damage that in time, unchecked and unsolved, develops into symptomatic cardiovascular issues in young adults (2). Childhood obesity has developed into a public health problem that necessitates ongoing attention and effort to slow down because of all the variables contributing to its worsening over time (3–7). Contributing factors that make up a constellation of obesity-related complications other than the metabolic syndrome (abdominal adiposity, high blood pressure, dyslipidemia, glucose intolerance) (1), are metabolic-associated fatty liver disease (MAFLD), polycystic ovary syndrome (PCOS), and sleep apnea (8). These are the primary metabolic factors responsible for the vascular walls’ gradual deterioration in children (9, 10).

In order to combat the long-term effects of childhood obesity with regard to cardiovascular complications, it’s important to assess the value of biomarkers that may amend our understanding of the individual metabolic status and prognosis.

Trimethylamine N-oxide (TMAO) is a metabolite that has received increased attention in the past years due to its link with metabolic syndrome (MetS) and cardiovascular disease (11). Significant data shows the link between serum TMAO concentrations and cardiovascular diseases and events in adults with cardio-metabolic comorbidities (12). Both serum levels of TMAO and its precursors have been shown to have prognostic value for cardiovascular events (13). We know less about the situation of metabolically healthy adults, and much less about children, regardless of their health status (14). However, studies that evaluated TMAO precursors showed that they are among the few serum markers with prognostic value for the subsequent development of cardiovascular disease in patients that are still free of cardiovascular events (14, 15).

Childhood obesity promotes the progression of subclinical atherosclerosis and arterial stiffness (16, 17). Due to the possible associations of serum TMAO with the progression of atherosclerosis in adults with cardio-metabolic risks (11), we decided to analyze the value of serum TMAO in our pediatric patients with chronic primary obesity and insulin resistance. Our aim is to evaluate any possible association between TMAO serum levels, adiposity, and vascular biomarkers investigating subclinical atherosclerosis and arterial stiffness. The vascular biomarkers included in this study are the carotid intima-media thickness (CIMT), the pulse wave velocity (PWV) and augmentation index (AIx), the peripheral and central blood pressure values.

TMAO is derived from dietary products like red meat, poultry, eggs, milk, and whole grains (18). These foods are rich in choline, L-carnitine, γ-butyrobetaine, trimethyl lysine, betaine, and δ-valerobetaine, which are precursors of TMAO (19). These products are digested and mostly absorbed through the intestinal mucosa (20). The excessive TMAO precursors are metabolized by the gut microbiota into trimethylamine (TMA). TMA is consecutively absorbed from the intestinal lumen into the bloodstream and oxidized by hepatic flavin monooxygenases (FMO1 and FMO3), thus resulting in TMAO (21).

Equally as important as the total intake of dietary TMAO precursors, if not more, is the type of predominant gut microbiota (22). The bacterial genera that have been shown to increase the production of TMA are *Bacteroidetes, Firmicutes, Proteobacteria*, and *Fusobacteria* (23). Probiotics and antibiotics may remodel the composition of the microbiota, thus reducing the quantities of TMAO substrate (24, 25). Obese individuals have been shown to have a gut microbiota reduced in diversity (26), with a relative abundance of *Bacteroidetes* and *Firmicutes* (27). Moreover, it seems that these microbes are involved in the energy acquisition for the host from the ingested foods (26, 28), probably via metabolites involved in the regulation of hunger, in order to meet their own needs by the host (28). Studies have shown that in germ-free conditions, obesity is reduced by the reduction of energy acquisition (29, 30). Studies focused on the impact of gut microbiota on obesity have attributed a low ratio of *Bacteroidetes* to *Firmicutes* to obesity. Intervention studies that found that high-calorie diets increased energy harvest while increasing *Firmicutes* and decreasing *Bacteroidetes* provide more support for this (31, 32). There is, therefore, significant data that suggests that a certain composition of gut microbiota is in fact a favorable factor for obesity and even insulin resistance and type 2 diabetes mellitus. In support of these claims are the studies on germ-free mice who despite being fed high-fat, sugar-rich diets, remained lean and did not develop insulin resistance, compared to normal-raised mice (30, 33).

What is TMAO’s role in the intricate mechanisms produced by MetS and insulin resistance? What we know so far is that individuals with metabolic disorders such as obesity, type 2 diabetes mellitus (34), MAFLD (22), chronic kidney disease (35), and gastrointestinal cancers (36–38), present higher levels of serum TMAO. The mechanisms and pathways in which TMAO, as a chemical, is involved in oxidative, osmotic, and hydrostatic stress are only partially understood (1). TMAO is involved in hepatic glucose metabolism by increasing gluconeogenesis and decreasing hepatic glucose transport (39) and in insulin resistance, possibly through TMAO-dependent higher levels of N-Nitroso Compounds that are promoters of insulin resistance (40, 41). TMAO is involved in the alteration of vascular health by multiple processes: the disturbance of bile acid metabolism, inhibition of the reverse transport of cholesterol, inducement of foam cell formation, activation of platelets, and overall oxidative stress and vascular inflammation (22, 42). These processes, along with other metabolic disturbances promoted by obesity and insulin resistance, directly promote the development of MAFLD (22), which is an entity directly associated with atherosclerosis (43).

Practically, the cycle starts with excessive diets that promote weight gain and the modification of gut microbiota that is both influenced by obesity and seems to favor obesity as well. The consequent elevation of serum metabolites like TMAO interferes with metabolic pathways which promote oxidative stress and inflammation. Obesity maintains the vicious cycle and, in time, these intricate pathophysiological mechanisms lead to the aggravation of metabolic status and vascular health. The younger the age at which these risk factors appear and the longer the evolution of obesity, the higher the long-term risk of developing metabolic and vascular complications.

The reason this study’s design includes non-invasive biomarkers of arterial dysfunction is that TMAO’s involvement in vascular wall pathogenesis is linked to superoxide anion-associated oxidative stress, which leads to the reduction of nitric oxide, concluding in an increase in the vascular tone. In addition to this oxidative stress, advanced glycation end products are involved in the formation of cross-links between collagen and other proteins involved in the structure of the arterial wall. The causal-effect connection, however, is not yet undoubtedly proven, as TMAO’s effects are not exclusive. These alterations are also linked to aging. Furthermore, it has been observed that TMAO serum levels positively correlate to age (44).

Evaluating subclinical atherosclerosis and arterial stiffness in children is not part of the routine. However, studies have shown that obese children present signs of a more advanced atherosclerosis process than their lean peers (45). This can be evaluated by measuring the CIMT (16, 46–49) and the PWV, a marker of arterial stiffness (49–52). Peripheral and central blood pressure measurements bring additional data on the status of vascular health. The CIMT is an ultrasound measurement of the distance between the smooth muscle cells of the media and the endothelial cells of the artery’s intima lining. It is a measure that reliably identifies the degree of generalized atherosclerosis and is frequently applied to adult patients undergoing cardiovascular diagnostic and risk assessment (46). It is a non-invasive indicator of subclinical atherosclerosis that can be helpful in kids who have cardiovascular risks (16). The elasticity of large arteries is described by the PWV. It essentially reconstructs the aortic pulse wave and provides an estimate of the pulse velocity through the arterial tree (50, 51). Due to its elevated values being associated with myocardial remodeling, PWV is a trustworthy predictor of cardiovascular events (53).

Therefore, this study assesses the value of TMAO as a serum biomarker in correlation to non-invasive markers like CIMT, PWV, and other vascular biomarkers, to try to enhance our understanding of the progression of atherosclerosis in obese children with and without insulin resistance.

## 2 Materials and methods

The study involved 70 children of both sexes, aged four to eighteen, and was conducted from November 2022 to May 2023 at the Pediatric Endocrinology Department of the “Pius Brinzeu” Emergency County Hospital in Timisoara.

The Ethics Committee for Scientific Research of the “Pius Brinzeu” Emergency County Hospital has approved the study (No. 349/15.11.2022) in accordance with the Helsinki Declaration. Informed consent was signed by each participant’s legal guardian after explanations regarding the medical procedures that would take place.

The participants were recruited from the patients addressed to our department with weight excess.

Inclusion criteria – all three criteria were mandatory:

■ chronic primary obesity (caused by the energy intake/expenditure imbalance and defined by the CDC as presenting a BMI > 95^th^ percentile) (54);
■ waist circumference (WC) > 90^th^ percentile, measured using a flexible, non-elastic anthropometric tape, positioned halfway between the lowest rib and the upper edge of the iliac crest, at the end of a normal exhalation (55);
■ diverse diet, including meat, fish, eggs, dairy products, and grains.

Exclusion criteria:

■ acute illness at the time of examination (56);
■ secondary causes of obesity (57);
■ type II diabetes mellitus;
■ impairment in kidney function or chronic kidney disease (GFR < 90 ml/min/1.73 m2, CKD EPI) (58);
■ medical history of chronic digestive diseases or acute digestive diseases within 3 months of serum TMAO determination;
■ use of antibiotics and probiotics within 2 months of serum TMAO determination (25);
■ constant exposure to cigarette smoke;
■ special diets: vegetarian/vegan, gluten-exclusion diet.

The Homeostatic Model Assessment for Insulin Resistance (HOMA-IR) was used to divide the 50 obese participants (BMI > the 95^th^ percentile) into two groups: obese with HOMA-IR ≥ cut-off values for individual Tanner development, and obese with HOMA-IR <cut-off values (see section 2.2. Biochemical assay). Twenty age-matched normal-weight children (BMI ranging between percentiles 5 and 85) were included in the control group. The same exclusion criteria were applied to them as well. These participants were volunteers selected from the families of the medical personnel in our clinic.

### 2.1 Physical examination

All participants were physically examined. We measured their weight, height, and waist circumference (WC). To calculate the Body Mass Index (BMI) value we used the formula BMI= Weight (kg)/Height^2^ (m) (54) and interpreted it by using the 2022 extended growth charts for BMI for age and sex (59). The waist-to-height ratio (WHR) was also calculated by using the formula WHR = WC (m)/Height (m) (60). WC values were interpreted by using the percentiles proposed by Zong et al. (54). Only children with a BMI > 95^th^ percentile and a WC > 90^th^ percentile were included in the study group. Tanner stages were determined for each participant (Tanner 1 represents pre-puberty; Tanner 2-4 represents puberty; Tanner 5 represents post-puberty) (61). The presence of acanthosis nigricans was noted (62). Post-pubertal girls were asked whether they had regular or irregular menses. Irregular menses were considered the menstrual cycles <21 or >35 days, or missing ≥ 3 menses in a row, in girls who had their menarche at least 2 years before (50).

### 2.2 Biochemical assay

Blood tests were taken within a week of the physical examination and scheduled between 7:30 and 8:30 a.m. Patients were asked to fast for at least 12 hours before giving blood. The serum parameters determinations were carried out in an accredited laboratory. Serum TMAO was determined through liquid chromatography-mass spectrometry (laboratory cut-off values: normal < 270 µg/l; borderline ≥270 µg/l, <380 µg/l; high ≥ 380 µg/l). Other serum parameters determined were insulin (chemiluminescence immunoassay), fasting glucose, uric acid, creatinine, lipid profile (total cholesterol, LDL-cholesterol, HDL-cholesterol, non-HDL-cholesterol), triglycerides (TG), aspartate aminotransferase (AST), alanine aminotransferase (ALT), and 25-OH-Vitamin D (spectrophotometry assay).

The HOMA-IR was calculated using the formula HOMA-IR = [Glucose (mg/dl) × Insulin (µU/mL)]/405 (63). Cut-off values for pre-pubertal children for insulin resistance detection were considered 2.3 (64) and for pubertal and post-pubertal children, 3.4 (64, 65).

The Visceral Adiposity Index (VAI) was calculated by sex-specific formulas: for males VAI = {WC/[39.68 + (1.88 × BMI)]} × (TG/1.03) × (1.31/HDL-c), and for females VAI = {WC/[36.58 + (1.89 × BMI)]} × (TG/0.81) × (1.52/HDL-c), with WC measured in cm, and TG and HDL-c in mmol/l (64, 66).

The triglyceride–glucose index (TyG index), a marker of muscular resistance to insulin, was calculated using the formula TyG index= Ln[(TG × Glucose/2], with TG and glucose measured in mg/dl (67).

### 2.3 Carotid intima-media thickness measurement

The Aixplorer MACH 30 echography device was used to ultrasonographically measure the carotid intima-media thickness (Country-SuperSonic Imagine, Aix-en-Provence, France). An experienced and certified sonographer has performed carotid ultrasonography on each patient included in this study. Depending on the amount of adipous tissue in each subject’s cervical area, the chosen linear transducer was either the SL 10-2 (2-10 MHz) or the SL 18-5 (5-18 MHz). The CIMT values can be calculated by the echography machine software automatically (SuperSonic Imagine, Aix-en-Provence, France). The images were acquired during the end-diastole as determined by the R wave on the electrocardiogram (68). Ten CIMT measurements were performed for each patient, five on each side, and the mean value was used in the study.

To enable a better view of the common right and left carotid arteries, the subject is requested to lie down and stretch their neck backward as far as possible. The appropriate ultrasound settings and ultrasound transducer are chosen by the examiner. Transversal scanning starts from the collarbone upward to locate the carotid bulb and the subsequent bifurcation of the common carotid into the internal and external carotids. After locating the carotid bulb, the examiner turns to longitudinal scanning. On this section, the carotid bulb appears on the left side of the screen. The measurements are made on the posterior carotid wall, at approximately 1-2 cm caudally from the bulb (69, 70). During the end-diastole, the image is frozen and the software performs the CIMT measurement in the region of interest selected by the examiner (71, 72).

### 2.4 Pulse wave analysis

The Mobil-O-Graph® 24 Hour ABPM oscillometric device (M26101200, IEM® GmbH, Stolberg, Germany) was used to perform the pulse wave analysis (PWA). The PWA includes the following parameters which represent markers of arterial stiffness: PWV (m/s), augmentation index (AIx, %), heart rate (HR), peripheral and central blood pressure (BP, mmHg). The mean arterial pressure (MAP, mmHg) and central pulse pressure (cPP, mmHg) are also calculated by the device. This device can be used in children older than three years old, according to the manufacturer. Details of the methodology and specifics of the device have been presented in our previous work (50, 73).

The patients were advised to not consume any caffeinated drinks for a day prior to the PWA measurements, to avoid exposure to cigarette smoke for at least 4 hours prior, and to make sure they slept for at least 8 hours the night before. Before performing the PWA measurements, the patients were explained in detail the whole procedure in order to make sure the psychological stress and the consequential potential raise in heart rate values are reduced. After the discussion, the participants were left to rest and relax for ten minutes. A single-point brachial measurement was performed for each participant. The appropriate cuff size was chosen from four options: extra small: 14-20 cm; small: 20-24 cm; medium: 24-32 cm; large: 32-38 cm. The cuff was placed on the left bare arm, with the artery symbol positioned on the region of the brachial artery. The participants were advised not to speak or move during the measurement and remain as relaxed as possible.

The device performs two measurements, divided by a thirty-second pause. Firstly, the peripheral BP values are obtained, and secondly, the PWV, AIx, HR, and central BP. Should the measurement be considered inaccurate by the software (in the case the patient was moving), we repeated it after a five-minute break.

### 2.5 Statistical analysis

Data was gathered using Microsoft Excel, and statistics were performed using MedCalc Statistical Software version 20.111 (MedCalc Software Ltd., Ostend, Belgium) and DATAtab: Online Statistics Calculator (Graz, Austria). The link between serum TMAO levels and clinical and paraclinical markers was the main focus of the analysis. The Shapiro-Wilk test was used to determine whether the data distribution was normal. Hence, we conducted the statistical tests that were appropriate for the level of normality of the data: means, student T-test, Pearson’s correlations for the normally distributed data, medians, Mann-Whitney test, and Spearman’s correlation for the non-normal variables. P-values lower than 0.05 were considered significant. When three groups were compared, for non-normal variables, Krussal-Wallis H-tests were carried out, with Dunn-Bonferroni tests for the two-by-two comparisons, and for normal variables, one-way ANOVA *post-hoc* tests with Bonferroni-corrected p-values were performed. Linear regression analyses were performed in order to investigate the influence of TMAO (independent variable) on the clinical and paraclinical markers (dependent variables), and multiple regressions for examining the reverse situation.

## 3 Results

The participants were divided into two groups of study: obese (*n*=50) and controls (*n*=20). The obese group was further divided into two groups:

■ obese with HOMA-IR ≥ cut-off values for individual pubertal development, n=21 (called further on “obese with IR”);
■ obese with HOMA-IR < cut-off values for individual pubertal development, n=29 (called further on “obese without IR”);

**Table 1** depicts how the participants were divided into study subgroups.

**Table 1:** Number of participants in each studies subgroup, by sex, age, and puberty development.

		Obese with IR (*n*=21)	Obese without IR (*n*=29)	Controls (*n*=20)
Sex	Male	13	18	10
	Female	8	11	10
Age	<12 y	10	12	7
	12-15 y	8	13	10
	> 15 y	3	4	3
Tanner stage	Tanner 1	8	7	6
	Tanner 2-4	6	13	9
	Tanner 5	7	9	5

Out of the 70 participants, 68.57% presented normal levels of serum TMAO (45.71% were obese and 22.85% were normal-weight), 20% presented borderline values (14.28% obese and 5.71%, normal-weight), and 11.43% presented high levels, all of which were obese (see **Table 2**).

**Table 2:** Data regarding the mean levels of TMAO and their distribution among participants.

serum TMAO categories	*n*			Mean TMAO (µg/l)	SD
	total	obese	controls		
Normal <270 µg/l	48	32	16	173.61	58.6
Borderline ≥ 270, <380 µg/l	14	10	4	325.34	36.54
High ≥ 380 µg/l	8	8	0	597.41	120.25

### 3.1 Characteristics of all subjects involved in the study

**Table 3** describes the general characteristics of the participants and the differences noted with regard to the studied parameters between obese subjects and controls. The clinical markers of adiposity (W, BMI, WC, WHR) were significantly higher in the obese subjects. All vascular markers, except for HR and cPP displayed significantly higher values in the obese. Blood parameters depicting IR confirmed extremely significantly higher values for HOMA-IR and fasting glucose in obese subjects, but not higher TyG index and VAI. The uric acid and creatinine were also significantly increased. Regarding the lipid fractions, the obese did not present dyslipidemic levels compared to the controls, in fact, the controls presented a significantly lower HDL-c and a significantly higher TC/HDL-c ratio. 25-OH-Vitamin D presented significantly lower levels in the obese. Serum TMAO median values were significantly higher in the obese subjects, compared to normal-weight ones (*p*=0.02), see **Supplementary Figure 1**.

**Table 3:** General descriptive data and comparisons between obese subjects and controls.

Parameter	Obese		Controls		*p*
	Mean/Median	SD	Mean/Median	SD	
Age (y)	11.92	3.46	11.7	3.33	0.8
W (kg)	76.18	25.36	43.3	12.7	**<0.001**
H (cm)	153.8	17.17	150.2	16.3	0.77
BMI (kg/m^2^)	31.36	6.14	18.51	2.25	**<0.001**
WC (cm)	98.06	15.81	65.65	8.01	**<0.001**
WHR	0.64	0.09	0.44	0.03	**<0.001**
CIMT (mm)	0.47	0.05	0.42	0.04	**<0.001**
PWV (m/s)	5.02	0.41	4.45	0.38	**<0.001**
AIx (%)	26.06	9.13	20.95	2.1	**0.04**
HR (b/min)	84.92	6.47	82.35	6.76	0.14
SBP (mmHg)	128.58	9.59	114.5	8.84	**<0.001**
DBP (mmHg)	82.48	7.76	76.95	7.06	**0.007**
MAP (mmHg)	105.53	7.36	95.73	7.43	**<0.001**
cSBP (mmHg)	116.26	10.4	100.85	10.54	**<0.001**
cDBP (mmHg)	72.3	8.83	69.05	8.44	0.16
cPP (mmHg)	93.15	12.11	41.8	6.61	**<0.001**
Serum TMAO (µg/l)	226	163.7	175.15	88.37	**0.02**
HOMA-IR	3.4	2.53	1.81	0.81	**<0.001**
TyG index	8.18	0.4	8.16	0.5	0.86
VAI	1.31	0.84	1.23	0.77	0.96
Fasting glucose (mg/dl)	80.95	8.38	76.22	7.46	**0.02**
uric acid (mg/dl)	5.06	1.5	3.78	0.72	**<0.001**
creatinine (mg/dl)	0.55	0.16	0.46	0.12	0.02
total cholesterol (mg/dl)	161.22	37.86	162.9	28.93	0.86
LDL-cholesterol (mg/dl)	95.4	29.81	104.4	27.3	0.24
HDL-cholesterol (mg/dl)	48	11.08	41.5	10.84	**0.02**
non-HDL-cholesterol (mg/dl)	113.22	34.59	121.45	30.3	0.35
Triglycerides (mg/dl)	100.74	37.54	97.4	51.35	0.76
TC/HDL-c ratio	3.45	0.81	4.1	0.95	**0.005**
TG/HDL-c ratio	2.25	1.09	2.47	1.34	0.46
AST (U/l)	26.94	10.25	22.6	8.35	0.09
ALT (U/l)	28.82	12.26	24	10.34	0.12
25-OH-Vitamin D (ng/ml)	19.8	6.63	24.2	8.82	**0.02**

*The significant results in this table were bolded.

No differences were detected when comparing obese participants vs. controls according to sex (*p*=0.44), and neither between obese boys vs. obese girls (0.32). The girls in the control group presented significantly lower TMAO levels compared to the boys in the control group (*p*=0.002) and also compared to the obese girls (*p*=0.0006). No differences were detected between obese boys vs. normal-weight boys (*p*=0.62). See **Supplementary Table 1**.

All subjects were divided into age and puberty-development subgroups. The Krussal-Wallis tests performed revealed that there are no significant differences in serum TMAO levels between children according to age groups (*p*=0.37), see **Supplementary Table 2**. The analysis also revealed no differences between the TMAO levels between subjects in different stages of puberty development (*p*=0.59), see **Supplementary Table 3**.

### 3.2 Characteristics of the obese group: does insulin resistance make a difference?

No differences between the boys and girls in the obese with IR group, nor the obese without IR were detected, and neither for the same-sex participants between the two groups, as depicted in **Table 4**.

**Table 4:** Differences between median TMAO values between sexes, for the obese participants (Mann-Whitney tests).

Sex	Serum TMAO median values		
	Obese with IR	Obese without IR	*p*
Male	226.9	218.5	0.56
Female	292.1	287	0.53
*p*	0.85	0.29	

The differences between the TMAO levels of the participants divided by age into three subgroups were close to meeting the significance threshold (*p*=0.05), with the TMAO levels of children under 12 years old being the highest (**Table 5**).

**Table 5:** Differences between median TMAO values between age groups, including the obese subjects (Krussal-Wallis H-test, Dunn-Bonferroni-Tests for the two-by-two comparisons).

	Median values				*p*		
	<12 y	12-15 y	≥15 y	H-test *p*	< 12 y vs. 12-15 y	<12 y vs. ≥15 y	12-15 y vs. ≥ 15 y
Serum TMAO	302.9	201.4	224.8	0.05	0.3	0.94	0.9

No differences were detected between the TMAO levels of the subjects with regard to their pubertal development. The highest median value was detected in the pre-pubertal subgroup, but the statistical difference threshold was not met upon comparing the data (**Table 6**).

**Table 6:** Differences between median TMAO values between stage development groups, including the obese subjects (Krussal-Wallis H-test, Dunn-Bonferroni-Tests for the two-by-two comparisons).

	Median values			H-test *p*	*p*		
	Tanner 1	Tanner 2-4	Tanner 5		Tanner 1 vs Tanner 2-4	Tanner 1 vs. Tanner 5	Tanner 2-4 vs. Tanner 5
Serum TMAO	302.9	213.8	267.8	0.8	0.15	0.85	0.21

**Table 7** describes the general characteristics of the obese participants with regard to clinical and paraclinical parameters and the differences noted with regard to the studied parameters between obese children with IR and without IR, respectively. Concerning clinical parameters, the subjects with IR presented significantly higher W, BMI, WC, and WHR, than did the obese without IR. All the vascular biomarkers measured showed significantly increased values in obese children with IR, except for HR and cSBP. Blood parameters depicting IR confirmed extremely significantly higher HOMA-IR (*p*<0.0001), serum insulin (*p* **<**0.0001), and fasting glucose (*p*=0.005) levels in obese with IR. However, the other two parameters suggestive for IR, TyG index and VAI, did not display significant increases in the obese with IR group. No significant differences were detected between the two groups with regard to the other blood tests, except for the ALT (*p*=0.0008). Although serum TMAO median values were higher in the obese with IR group (η=294.48 µg/l vs. η=271.83 µg/l), the threshold for statistical significance was not met (p=0.63).

**Table 7:** Descriptive and comparative data of the obese subjects (T-student tests and Mann-Whitney tests, according to the normality of the data).

Parameter	Obese with IR		Obese without IR		*p*
	Mean/Median	SD	Mean/Median	SD	
Age (y)	11.7	3.84	12	3.2	0.69
W (kg)	83.56	30.17	70.83	20.11	**0.04**
H (cm)	153	0.19	154	0.16	0.85
BMI (kg/m^2^)	34.48	7.29	29.09	3.9	**0.001**
WC (cm)	103.33	19.06	94.24	11.92	**0.04**
WHR	0.67	0.1	0.61	0.06	**0.01**
CIMT (mm)	0.49	0.04	0.46	0.04	**0.02**
PWV (m/s)	5.18	0.42	4.91	0.37	**0.02**
AIx (%)	31.23	7.55	22.31	8.39	**0.0003**
HR (b/min)	85.57	6.54	84.44	6.48	0.55
SBP (mmHg)	131	9.79	125.65	8.45	**0.01**
DBP (mmHg)	85.47	6.52	82	7.95	**0.01**
MAP (mmHg)	109.04	6.78	102.98	6.77	**0.003**
cSBP (mmHg)	119.61	11.82	113.82	8.66	0.05
cDBP (mmHg)	77	8.33	68.89	7.64	**0.0008**
cPP (mmHg)	96.5	16.7	91.36	7.01	**0.02**
Serum TMAO (µg/l)	294.48	175.3	271.83	157.23	0.63
HOMA-IR	4.6	2.9	2.05	0.7	**< 0.0001**
TyG index	8.28	0.37	8.1	0.4	0.11
VAI	1.51	0.71	1.38	0.93	0.57
Serum Insulin (µU/mL)	23.8	13.66	11.39	3.64	**<0.0001**
Fasting glucose (mg/dl)	79.61	6.66	73.75	7.11	**0.005**
uric acid (mg/dl)	5.3	1.64	4.8	1.38	0.23
creatinine (mg/dl)	0.53	0.17	0.56	0.15	0.49
total cholesterol (mg/dl)	159.28	47.04	162.62	30.37	0.76
LDL-cholesterol (mg/dl)	90.45	34.67	98.96	25.77	0.14
HDL-cholesterol (mg/dl)	48.38	13.54	47.72	9.15	0.83
non-HDL-cholesterol (mg/dl)	110.9	41.05	114.86	29.71	0.11
Triglycerides (mg/dl)	105.33	36.37	97.41	38.65	0.46
TC/HDL-c ratio	3.36	0.8	3.5	0.81	0.99
TG/HDL-c ratio	2.36	1.14	2.15	1.06	0.51
AST (U/l)	27.9	7.58	24	11.89	0.1
ALT (U/l)	32	13.57	23	9.17	**0.0008**
25-OH-Vitamin D (ng/ml)	17.1	7.21	20.12	6.28	0.43

*The significant results in this table were bolded.

### 3.3 Correlation analysis

Spearman’s correlation analysis between the clinical and paraclinical parameters and the serum TMAO levels showed multiple significant correlations. Age does not correlate to TMAO levels. The BMI, WC, and WHR significantly correlate to TMAO levels (**Table 8**). Regarding the vascular markers, significant moderate correlations were detected between CIMT (**Supplementary Figure 2**), PWV (**Supplementary Figure 3**), peripheral BP values, and serum TMAO levels (**Table 9**). Of the blood tests analyzed, only fasting glucose showed a negative moderate correlation to TMAO, while TG/HDL-c ratio and ALT revealed moderate positive correlations to TMAO. Age did not reveal a positive correlation to TMAO (**Table 10**).

**Table 8:** Correlations between serum TMAO and the clinical parameters (Spearman’s correlations).

Parameters		Serum TMAO	
		All subjects	Obese subjects
Age (y)	ρ	-0.001	-0.19
	*p*	0.98	0.17
W (kg)	ρ	0.17	-0.08
	*p*	0.15	0.55
BMI (kg/m^2^)	ρ	**0.27**	0.06
	*p*	**0.02**	0.65
WC (cm)	ρ	**0.25**	0.004
	*p*	0.03	0.99
WHR	ρ	0.34	0.25
	*p*	0.004	0.07

*The significant results in this table were bolded.

**Table 9:** Correlations between serum TMAO and the vascular parameters (Spearman’s correlations).

Parameters		Serum TMAO	
		All subjects	Obese subjects
CIMT (mm)	ρ	**0.48**	**0.29**
	*p*	**<0.001**	**0.04**
PWV (m/s)	ρ	**0.41**	**0.4**
	*p*	**0.0004**	**0.003**
AIx (%)	ρ	0.18	0.15
	*p*	0.12	0.29
HR (b/min)	ρ	0.14	**0.43**
	*p*	0.21	**0.001**
SBP (mmHg)	ρ	**0.3**	0.18
	*p*	**0.009**	0.19
DBP (mmHg)	ρ	**0.35**	**0.28**
	*p*	**0.002**	**0.04**
MAP (mmHg)	ρ	**0.35**	0.25
	*p*	**0.003**	0.07
cSBP (mmHg)	ρ	0.3	0.2
	*p*	0.009	0.15
cDBP (mmHg)	ρ	0.13	0.08
	*p*	0.25	0.57
cPP (mmHg)	ρ	0.27	0.11
	*p*	0.02	0.42

*The significant results in this table were bolded.

**Table 10:** Correlations between serum TMAO and the blood parameters (Spearman’s correlations).

Parameters		Serum TMAO	
		All subjects	Obese subjects
HOMA-IR	ρ	0.14	-0.07
	*p*	0.22	0.6
TyG index	ρ	0.12	-0.002
	*p*	0.3	0.98
VAI	ρ	0.2	0.16
	*p*	0.09	0.24
Insulin (µU/mL)	ρ	0.15	-0.05
	*p*	0.21	0.72
Fasting glucose (mg/dl)	ρ	**-0.27**	**-0.3**
	*p*	**0.02**	**0.02**
uric acid (mg/dl)	ρ	0.01	-0.1
	*p*	0.9	0.46
creatinine (mg/dl)	ρ	-0.1	-0.2
	*p*	0.38	0.16
total cholesterol (mg/dl)	ρ	0.06	0.09
	*p*	0.61	0.52
LDL-cholesterol (mg/dl)	ρ	0.13	0.16
	*p*	0.26	0.24
HDL-cholesterol (mg/dl)	ρ	-0.17	-0.25
	*p*	0.15	0.07
non-HDL-cholesterol (mg/dl)	ρ	0.13	0.18
	*p*	0.27	0.21
Triglycerides (mg/dl)	ρ	0.18	0.07
	*p*	0.12	0.6
TC/HDL-c ratio	ρ	0.14	0.22
	*p*	0.24	0.12
TG/HDL-c ratio	ρ	**0.24**	**0.17**
	*p*	**0.04**	**0.23**
AST (U/l)	ρ	0.21	0.03
	*p*	0.07	0.83
ALT (U/l)	ρ	**0.25**	0.07
	*p*	**0.03**	0.59
25-OH-Vitamin D (ng/ml)	ρ	-0.08	-0.03
	*p*	0.47	0.81

*The significant results in this table were bolded.

Furthermore, the correlation analysis between the analyzed parameters and the TMAO levels focused only on obese subjects, revealed moderate positive correlations between serum TMAO and CIMT, PWV, HR, and DBP (**Table 9**). Fasting glucose remains the only blood parameter to show a significant negative correlation to TMAO (**Table 10**).

### 3.4 Regression analysis

In order to measure the influence of serum TMAO on the vascular markers, linear regressions were performed between TMAO (independent variable) and each clinical, vascular, and blood parameter (dependent variable). According to our analysis, serum TMAO levels independently predict the values of, W, BMI, WC, WHR, CIMT, PWV, SBP, central BP levels, HR, and ALT (**Table 11**).

**Table 11:** Linear regressions depicting the relationship between serum TMAO (independent variable) and the analyzed parameters.

Dependent variable	Regression model			Regression coefficients and significance				
	F	R^2^	Variance %	B coefficient	β coefficient	Std. error	t	*p*
W	4.39	0.06	6.07	55.82	0.25	0.02	2.1	**0.04**
BMI	8.84	0.12	11.5	23.26	0.34	0.01	2.97	**0.004**
WC	7.86	0.006	10.4	77.97	0.32	0.02	2.81	**0.006**
WHR	9.89	0.13	12.7	0.51	0.36	0	3.15	**0.002**
CIMT	22.81	0.25	25.12	0.42	0.5	0	4.78	**<0.001**
PWV	18.76	0.22	21.62	4.49	0.46	0	4.33	**<0.001**
AIx	2.32	0.03	3.29	21.77	0.18	0.01	1.52	0.13
SBP	13.41	0.16	16.47	116.97	0.41	0.01	3.66	**<0.001**
DBP	9.28	0.12	12.01	76.36	0.35	0.01	3.05	**0.003**
MAP	15.22	0.18	18.29	96.66	0.43	0.01	3.9	**<0.001**
cSBP	13.81	0.17	16.88	103.36	0.41	0.01	3.72	**<0.001**
cDBP	4.18	0.06	5.79	67.87	0.24	0.01	2.04	**0.04**
cPP	8.88	0.18	11.55	58.81	0.34	0.02	2.98	**0.004**
HR	5.9	0.08	7.99	81.1	0.28	0.01	2.43	**0.01**
HOMA-IR	3.03	0.04	4.27	2.16	0.21	0	1.74	0.08
fasting glucose	3.37	0.05	4.72	80.43	-0.22	0.01	-1.84	0.7
VAI	3.16	0.04	4.44	1.2	0.21	0	1.78	0.08
TyG index	0.88	0.01	1.28	8.09	0.11	0	0.94	0.35
total cholesterol	0.05	-0.01	0.08	163.3	-0.03	0.03	-0.23	0.82
HDL-c	1.65	0.02	2.36	49.01	-0.15	0.01	-1.28	0.2
LDL-c	0.02	-0.01	0.02	98.7	-0.02	0.02	-0.13	0.9
triglycerides	1.01	0.01	1.46	91.48	0.12	0.03	1	0.31
non-HDL-c	0.04	-0.01	0.05	114.29	0.02	0.03	0.19	0.84
TG/HDL-c	2.23	0.03	3.17	1.97	0.18	0	1.49	0.14
TC/HDL-c	0.66	0.01	0.96	3.49	0.01	0	0.81	0.42
uric acid	0.37	0.01	0.54	4.52	0.07	0	0.61	0.54
creatinine	1.49	0.02	2.14	0.56	-0.15	0	-1.22	0.22
AST	0.42	0.01	0.62	24.42	0.08	0.01	0.65	0.51
ALT	4.58	0.06	6.31	22.52	0.25	0.01	2.14	**0.03**
25-OH-Vitamin D	<0.01	<0.01	0.01	21.16	-0.01	0.01	-0.06	0.95

*The independent variable for all the linear regression models is serum TMAO.

*The significant results in this table were bolded.

When adding other clinical and sanguine markers (HOMA-IR, fasting glucose, lipid fractions, triglycerides, uric acid, creatinine, AST, ALT, and 25-OH-Vitamin D) next to TMAO as influencers of the values of vascular parameters, the following results are observed.

#### 3.4.1 TMAO - CIMT

TMAO remains a significant predictor of CIMT, even when adding BMI, WC, and WHR in the regression model. When adding the pubertal development (Tanner stages) into the model (F=20.68, *p*<0.001, R^2^ = 0.73), only TMAO (β=0.37, *p*<0.001) remains a significant predictor, while BMI (β=0.27, *p*=0.08), WC (β=0.44, *p*=0.05), and WHR (β=0.3, *p*=0.11) do not. In this model, we observed a β=0.37 and *p*<0.001 for Tanner stage 5. When adding age into the model, significance does not change (TMAO and Tanner’s stage remain significant), however, age does not represent a significant predictor (F=18.08, *p*<0.001, R^2^ = 0.73).

The multiple regression including TMAO and the blood parameters, shows that TMAO remains a significant predictor of CIMT, along with the uric acid, creatinine, and ALT. See **Table 12**.

**Table 12:** Multiple regression analysis between TMAO, clinical markers (BMI, WC, WHR) and blood markers (HOMA-IR, fasting glucose, lipid fractions, triglycerides, uric acid, creatinine, AST, ALT, and 25-OH-Vitamin D) as influencers of the values of the vascular parameters.

Dependent variable	Independent variables	Significant independent variables	β	*p*	Regression model
CIMT	TMAO + clinical markers	TMAO	0.34	<0.001	F=30.38, *p*<0.001, R^2^ = 0.65
		BMI	0.42	0.01	
		WC	0.72	<0.001	
		WHR	0.62	<0.001	
	TMAO + blood markers	TMAO	0.41	<0.001	F=5.58, *p*<0.001, R^2^ = 0.56
		uric acid	0.24	0.03	
		creatinine	0.3	0.009	
		ALT	0.28	0.02	
PWV	TMAO + clinical markers	TMAO	0.29	0.002	F=21.58, *p*<0.001, R^2^ = 0.57
		BMI	0.41	0.03	
		WC	0.55	0.009	
		WHR	0.39	0.02	
	TMAO + blood markers	TMAO	0.38	0.001	F=21.58, *p*<0.001, R^2^ = 0.57
		HOMA-IR	0.3	0.01	
		creatinine	0.3	0.01	
		AST	0.27	0.02	
AIx	TMAO + clinical markers	BMI	0.65	0.008	F=7.54, *p*<0.001, R^2^ = 0.32
		WHR	0.58	0.008	
SBP	TMAO + clinical markers	TMAO	0.21	0.01	F=30.05, *p*<0.001, R^2^ = 0.65
		BMI	0.54	0.02	
		WC	0.61	0.02	
		WHR	0.51	0.001	
	TMAO + blood markers	TMAO	0.24	0.002	F=5.48, *p*<0.001, R^2^ = 0.56
		HOMA-IR	0.45	<0.001	
		creatinine	0.26	0.02	
DBP	TMAO + clinical markers	TMAO	0.22	0.04	F=8.14, *p*<0.001, R^2^ = 0.3
	TMAO + blood markers	TMAO	0.24	0.04	F=3.91, *p*<0.001, R^2^ = 0.48)
		ALT	0.29	0.03	
MAP	TMAO + clinical markers	TMAO	0.24	0.004	F=28.14, *p*<0.001, R^2^ = 0.63
		BMI	0.57	0.002	
		WC	0.56	0.004	
		WHR	0.51	0.002	
	TMAO + blood markers	TMAO	0.27	0.008	F=6.21, *p*<0.001, R^2^ = 0.59
		HOMA-IR	0.4	<0.001	
		creatinine	0.23	0.03	
cSBP	TMAO + clinical markers	TMAO	0.22	0.01	F=23.54, *p*<0.001, R^2^ = 0.59
		BMI	0.39	0.03	
		WC	0.67	0.001	
		WHR	0.45	0.007	
	TMAO + blood markers	TMAO	0.26	0.02	F=4.05, *p*<0.001, R^2^ = 0.48
		HOMA-IR	0.44	<0.001	
cDBP	TMAO + clinical markers	BMI	0.48	0.04	F=7.83, *p*<0.001, R^2^ = 0.33
		WC	0.55	0.03	
		WHR	0.69	0.002	
	TMAO + blood markers	HOMA-IR	0.41	0.002	F=3.3, *p*=0.001, *R^2^ = *0.43
cPP	TMAO + clinical markers	BMI	0.35	0.02	F=37.59, *p*<0.001, R^2^ = 0.7
	TMAO + blood markers	TMAO	0.21	0.04	F=5.45, *p*<0.001, R^2^ = 0.56
		HOMA-IR	0.36	0.002	
		fasting glucose	-0.31	0.006	
		uric acid	0.24	0.03	

Only the significant results are presented in the table.

#### 3.4.2 TMAO – PWV

TMAO remains a significant predictor of PWV when adding BMI, WC, and WHR in the regression model. When adding the pubertal development (Tanner stages) into the model (F=11.15, *p*<0.001, R^2^ = 0.59), WC (β=0.6, *p*=0.03) and TMAO (β=0.29, *p=*0.002) remain significant predictors, while BMI (β=0.32, *p*=0.1) and WHR (β=0.37, *p*=0.11) and Tanner stages do not. When adding age into the model (F=24.55, *p*<0.001, R^2^ = 0.7), significance changes and only TMAO remains a significant predictor (β=0.29, *p=*0.002).

The multiple regression including TMAO and the blood parameters shows that TMAO remains a significant predictor of PWV, along with HOMA-IR, creatinine, and AST. See **Table 12**.

#### 3.4.3 TMAO – AIx

The multiple regression model including TMAO and clinical parameters showed that while the BMI and WHR are significant predictors of AIx, WC (β=0.34, *p*=0.19) and TMAO (β=0.06, *p*=0.58) are not. Only the BMI (β=0.59, *p*=0.02) remains significant after adding the pubertal development (Tanner stages) into the model (F=4.38, *p*<0.001, R^2^ = 0.36). The same happens when we add age into the model (F=3.86, *p*<0.001, R2 = 0.37) – only the BMI remains a significant predictor of AIx (β=0.6, *p*=0.02).

The multiple regression including TMAO and the blood parameters (F=2.06, *p*=0.001, R^2^ = 0.32) shows that TMAO does not remain a significant predictor of AIx (β=0.03, *p*=0.79), and neither do any of the other blood parameters. See **Table 12**.

#### 3.4.4 TMAO – SBP

TMAO remains a significant predictor of SBP when adding BMI, WC, and WHR in the regression model.When adding the pubertal development (Tanner stages) into the model (F=14.24, *p*<0.001, R^2^ = 0.65), the significance does not change, BMI (β=0.56, *p*=0.03), WC (β=0.63, *p*=0.01), WHR (β=0.53, *p*=0.01), and TMAO (β=0.2, *p=*0.01) remain significant predictors, while Tanner stages do not. When adding age into the model (F=12.81, *p*<0.001, R^2^ = 0.66), significance does not change, TMAO, BMI, WC, and WHR remain significant predictors, while age and puberty development do not.

The multiple regression including TMAO and the blood parameters shows that TMAO remains a significant predictor of SBP, along with HOMA-IR, and creatinine. See **Table 12**.

#### 3.4.5 TMAO – DBP

TMAO remains the only significant predictor of DBP when adding BMI, WC, and WHR in the regression model. When adding the pubertal development (Tanner stages) into the model (F=4.28, *p*<0.001, R^2^ = 0.36), the significance does not change – only TMAO remains a significant predictor (β=0.23, *p=*0.04). Tanner stages do not significantly influence DBP. When adding age into the model (F=3.95, *p*<0.001, R^2^ = 0.37), TMAO remains a significant predictor (β=0.24, *p=*0.04), while age and puberty development do not.

The multiple regression including TMAO and the blood parameters shows that TMAO remains a significant predictor of DBP, as well as ALT. See **Table 12**.

#### 3.4.6 TMAO – MAP

TMAO remains a significant predictor of MAP when adding BMI, WC, and WHR in the regression model. When adding the pubertal development (Tanner stages) into the model (F=13.68, *p*<0.001, R^2^ = 0.64), the significance does not change, BMI (β=0.56, *p*=0.003), WC (β=0.63, *p*=0.01), WHR (β=0.54, *p*=0.01), and TMAO (β=0.24, *p=*0.006) remain significant predictors, while Tanner stages do not. When adding age into the model (F=11.96, *p*<0.001, R^2^ = 0.64), BMI (β=0.56, *p*=0.004) and TMAO (β=0.24, *p*=0.007) remain significant predictors, while WC, WHR, age, and puberty development do not.

The multiple regression including TMAO and the blood parameters shows that TMAO remains a significant predictor of MAP, as well as HOMA-OR, and creatinine. See **Table 12**.

#### 3.4.7 TMAO – cSBP

TMAO remains a significant predictor of cSBP when adding BMI, WC, and WHR in the regression model. When adding the pubertal development (Tanner stages) into the model (F=11.6, *p*<0.001, R^2^ = 0.6), the significance does not change, BMI (β=0.44, *p*=0.02), WC (β=0.76, *p*=0.006), WHR (β=0.55, *p*=0.02), and TMAO (β=0.22, *p=*0.02) remain significant predictors, while Tanner stages do not. When adding age into the model (F=10.76, *p*<0.001, R^2^ = 0.62), significance does not change, all markers remain significant predictors, while age and puberty development do not.

The multiple regression including TMAO and the blood parameters shows that TMAO remains a significant predictor of cSBP), as well as HOMA-OR. See **Table 12**.

#### 3.4.8 TMAO – cDBP

The multiple regression model including TMAO and clinical parameters (F=7.83, *p*<0.001, R^2^ = 0.33), showed that while BMI, WC, WHR, are significant predictors of cDBP, TMAO does not (β=0.15, *p*=0.18). BMI, WC, and WHR remain significant after adding the pubertal development (Tanner stages) into the model (F=4.17, *p*<0.001, R^2^ = 0.35). When adding age into the model (F=3.66, *p*<0.001, R^2^ = 0.35), only BMI and WHR remain significant predictors of cDBP, while TMAO, age and puberty development do not.

The multiple regression including TMAO and the blood parameters shows that TMAO does not remain a significant predictor of cDBP (β=0.05, *p*=0.68), but HOMA-IR does. See **Table 12**.

#### 3.4.9 TMAO – cPP

The multiple regression model including TMAO and clinical parameters showed that only the BMI represents a significant predictor of cPP, and TMAO, WC, and WHR do not. Only the BMI (β=0.43, *p*=0.01) remains significant after adding the pubertal development (Tanner stages) into the model (F=18.99, *p*<0.001, R^2^ = 0.71). When adding age into the model (F=17.18, *p*<0.001, R^2^ = 0.72), BMI (β=0.4, *p*=0.01) and WC (β=0.71, *p*=0.03) are observed as predictors of cPP, while TMAO, age and puberty development do not. See **Table 12**.

The multiple regression including TMAO and the blood parameters shows that TMAO remains a significant predictor of cPP, along with HOMA-IR, fasting glucose, and uric acid. See **Table 12**.

A logistic regression analysis was performed to examine the influence of TMAO on the presence of acanthosis nigricans. The model was statistically significant (Chi2(1)=5.69, *p*=0.01), and showed that an increase in TMAO is associated with an increase in the probability for the presence of acanthosis nigricans, *p*=0.03, B=0, Odds Ratio=1).

In contrast, we performed regression analyses in order to detect if any of the clinical and blood parameters exert an influence on the serum TMAO levels.

Although if analyzed separately, through linear regressions, the clinical markers of adiposity have a significant influence on TMAO levels, W (F=4.39, R^2^ = 0.06, β=0.25, *p*=0.04), BMI (F=8.84, R^2^ = 0.12, β=0.34, *p*=0.004), WC (F=7.89, R^2^ = 0.1, β=0.32, *p*=0.006), WHR (F=9.89, R^2^ = 0.13, β=0.36, *p*=0.002), the multiple regression analysis investigating the same markers as independent predictors for serum TMAO (dependent variable) showed that the parameters explained 13.6% of the variance in TMAO and the ANOVA analysis revealed that this value was significantly different from zero (F=2.56, *p*=0.04, R^2^ = 0.14). However, none of the parameters were confirmed as significant independent predictors: W (β=0.39, *p*=0.68), BMI (β=-0.11, *p=*0.88), WC (β=-0.45, *p*=0.65), WHR (β=0.62, *p=*0.49).

Linear regressions showed that age does not influence the values of TMAO (F=0.1, R^2^ =-0.01, β=-0.04, *p*=0.75), and neither do Tanner stages (F=0.5, *p*=0.73, R^2^ = 0.03), nor sex (F=0.42, *p*=0.51, R^2^ = 0.01). The presence of acanthosis nigricans exerts a significant influence on the levels of TMAO (F=5.72, R^2^ = 0.08, β=0.28, *p*=0.02).

The multiple regression model examining the influence of the blood parameters investigating IR (HOMA-IR, fasting glucose, VAI, and TyG index) as independent predictors for serum TMAO (dependent variable), showed that these variables explained only 14.53% of the variance in TMAO, and this value was statistically different from zero (F=2.79, *p*=0.03, R^2^ = 0.15). HOMA-IR (β=0.26, *p*=0.03) and fasting glucose (β=-0.28, *p*=0.04) were confirmed as independent significant predictors of TMAO, while TyG index (β=0.03, *p*=0.88) and VAI (β=0.12, *p*=0.51) were not.

The multiple regression model involving total cholesterol, HDL-c, LDL-c, triglycerides, non-HDL-c, TG/HDL-c ratio, and TC/HDL-c ratio as independent predictors of TMAO did not reveal significant results (F=0.59, *p*=0.76, R^2^ = 0.06). Neither did the linear regression analysis performed separately for each lipid fraction and ratio.

The multiple regression model examining the influence of uric acid, creatinine, and transaminase levels (AST, ALT) on TMAO levels also showed insignificant results (F=1.94, *p*=0.11, R^2^ = 0.11). Linear regression analysis showed that of these markers, only ALT is a significant predictor of TMAO levels (F=4.58, R^2^ = 0.06, β=0.25, *p*=0.03).

The linear regression examining the influence of 25-OH-Vitamin D on TMAO levels showed that the former is not a significant predictor (F=0.23, *p*=0.63, R^2^ =-0.02).

### 3.5 Serum TMAO in relation to the menstrual cycle regularity

Out of 29 girls, 14 have finalized their puberty. Seven girls reported regular menses, and seven, irregular ones. We performed one-way ANOVA *post-hoc* analyses with regard to the possible differences in TMAO levels, clinical markers of adiposity (BMI, WC, WHR), and HOMA-IR between three subgroups: girls who have not had their menarche, girls with regular, and with irregular menses. Although the girls with irregular menses presented the highest mean values for TMAO levels and the most significant weight excess, the significance threshold was not met (**Table 13**).

**Table 13:** One-way ANOVA posthoc tests comparing data regarding girls’ menstrual cycles (the two-by-two comparisons present Bonferroni-corrected p-values).

	Mean values			ANOVA *p*	*p*		
	Without menarche	Regular menses	Irregular menses		without menarche vs. regular menses	Without menarche vs. irregular menses	Regular menses vs irregular menses
Serum TMAO	242.8	157.71	309.09	0.18	0.68	1	0.21
BMI	23.71	28.59	34.59	**0.01**	0.44	**0.008**	0.39
WC	77.87	91.57	105.14	**0.01**	0.34	**0.009**	0.52
WHR	0.55	0.56	0.65	0.24	1	0.32	0.54
HOMA-IR	2.62	2.91	2.83	0.93	1	1	1

*The significant results in this table were bolded.

*p=1 for the two-by-two comparisons represents the maximum p-value after the Bonferroni correction.

Analyzing only the obese girls, we observed that out of 19 obese girls, 11 have had their menarches. Four girls reported regular menses and six, irregular menses. Similar ANOVA *post-hoc* analyses to those depicted in **Table 10** were performed in order to detect any differences within the obese group. Although TMAO levels remain the highest in obese girls with irregular menses, the significance threshold was still not met (**Supplementary Table 4**).

## 4 Discussion

This research provides insight into the connections between the gut-derived metabolite, serum TMAO, and the early vascular alterations linked to obesity in children. Obesity is a promoter of an earlier progression of atherosclerosis and arterial stiffness (16, 17, 46–51, 53). Using non-invasive biomarkers like the carotid intima-media thickness and pulse wave velocity to detect early functional and structural modifications of the arterial walls could be useful long-term in these children (16, 50, 73). Showing a connection to a marker like TMAO, which has received much attention in the last decades concerning its role in numerous pathologies (74), is important.

Numerous studies on animal models have shown intricate metabolic connections between TMAO and insulin resistance, and atherosclerosis respectively, connecting diet, alterations in gut microbiota, and cardio-metabolic diseases (75–81). In recent years, TMAO and its precursors have gained increasing attention in studies involving human adults, as relevant data shows involvement in the pathophysiology of disorders promoting insulin resistance (82–85), atherosclerosis (86–89), the risk of developing chronic kidney disease (90, 91), CV disorders (92–94), MAFLD (95–97), psoriasis (98), cancer (99), and even Alzheimer’s disease (100). Data regarding the roles of TMAO in children are scarce, to say the least. Most of the existing studies focus on linking serum or urinary TMAO levels or its precursors to dietary habits (101–103). The few studies that have focused on linking TMAO to certain pathologies in children have focused on chronic kidney disease (104) or the CV risk in children with early-stage chronic kidney disease (105), autism spectrum disorders (106, 107), asthma (108, 109), phenylketonuria (110). We identified one study on pre-pubertal obese children with weight excess that showed lifestyle intervention resulting in weight loss reduces TMAO levels significantly (111). Given the lack of data regarding TMAO and vascular damage in obese children, the present study focuses on detecting possible links between serum TMAO and clinical, vascular markers, and blood parameters in this type of individuals.

Our findings provide compelling evidence supporting the association between serum TMAO and obesity in children. We observed significantly elevated levels of TMAO in obese children compared to those with normal weight. Using the cut-off values for serum TMAO proposed by the laboratory, recognizing that these values were intended for adults, the study showed that approximately 68% of the participants had normal TMAO levels, 20% had borderline values, and 11% had high levels. All individuals with high TMAO levels were obese. Furthermore, TMAO levels presented moderate positive correlations to BMI, WC, and WHR. Hence, the more significant the weight excess, especially central obesity, the higher the TMAO values. Barrea et al. have linked the increase in TMAO levels to the severity of obesity in adults (112). The regression analysis revealed that serum TMAO levels independently predicted BMI, WC, and WHR. On the other hand, the regression analysis investigating the influence of clinical markers of adiposity on TMAO levels yielded mixed results. While the markers were found to have a significant influence on TMAO levels when analyzed individually, the multiple regression analysis showed that these parameters explained only a small portion of the variance in TMAO, and none of them emerged as significant independent predictors. The elevated levels of TMAO observed in obese children could most likely be attributed to alterations in gut microbiota composition and function, which have been previously implicated in the pathogenesis of obesity (113). Altered brain-gut-microbiota interactions fuel dysregulated eating behavior and obesity in a stubborn cycle. The interplay between diet, gut signals, inflammation, and disrupted brain balance drives a preference for high-calorie foods, reinforcing gut dysbiosis (114). Early-life gut imbalance, often due to antibiotics, increases short-chain fatty acid production, reshaping liver lipid metabolism and promoting obesity (115). This dysbiosis, linked to reduced microbiota diversity (116), affects immunity (117) and escalates cardio-metabolic risks (118, 119). An intervention study on obese children, regardless of the cause, found that a diet rich in non-digestible carbs improved microbiota composition, reducing harmful metabolite-producing microbes (120).

In this study, the obese also displayed significantly higher CIMT, PWV, AIx, peripheral BP, and central SBP, but not higher HR or central DBP than controls. These results suggest a close association between obesity and adverse changes in vascular function and are in line with previous studies (16, 17, 46–51, 53). Significant moderate correlations were observed between TMAO and vascular markers such as CIMT, PWV, and peripheral BP levels. Moreover, the regression linear analysis revealed that serum TMAO independently predicts most vascular markers: CIMT, PWV, systolic BP, and central BP levels. We performed multiple regressions in order to see if TMAO remains a significant predictor of the vascular parameters after adding the clinical markers of adiposity, age, puberty development, or other blood tests into the models – results will be discussed further on.

Andraos et al.’s study on 1166 children and 1324 adults presents mixed findings regarding TMAO and atherosclerosis. Their research associates TMAO precursors, rather than TMAO itself, with cardiovascular traits and inflammatory markers in a population-based sample, encompassing CIMT, PWV, and BP levels (121). In adults, TMAO has been linked to increased CIMT. Randrianarisoa et al. found that elevated TMAO levels correlated with increased CIMT, even after accounting for established risk factors. Weight loss intervention was connected to CIMT reduction, particularly when TMAO levels dropped significantly (122). While CIMT reversibility after intervention is limited due to atherosclerosis’s nature, the possibility exists in early stages, holding significance for obese children with accelerated atherosclerosis (122).

Regarding arterial stiffness, Brunt et al. investigated the connection between TMAO, aortic stiffness, and blood pressure, showing higher TMAO levels in older adults, correlating with increased carotid-femoral PWV, regardless of traditional risk factors. However, this link diminished when age was factored in. Mouse experiments mirrored these findings, with TMAO supplementation raising aortic PWV. This suggests potential for preventing early aortic stiffening in young adults with elevated TMAO and therapeutic options for older adults, though human studies are required for validation (123). In our study, TMAO levels did not correlate with age, the analysis did not reveal significant differences in TMAO levels across the three subgroups divided by age (<12 y, 12 to15 y, and ≥15 y), and age was not confirmed as an independent predictor for TMAO. However, not only did TMAO still correlate with PWV and peripheral BP levels, but it was also confirmed as an independent predictor of PWV, peripheral BP, and central SBP levels, even after adding BMI, WC, WHR, puberty development, and age into the regression model. Although the linear regressions showed TMAO as a significant predictor of cDBP and cPP, after adding the clinical markers of adiposity, puberty development, and age into the model, the significance was lost. However, with regard to age, other studies have confirmed the fact that older adults present higher circulating TMAO, but the mechanisms behind this are not elucidated (124–126). Brunt et al. have stipulated, however, that the link to age-related vascular endothelial dysfunction may occur due to a decrease in the availability of nitric oxide, caused by oxidative stress (126, 127). It is likely that the connection between TMAO and this age-related vascular dysfunction is not yet evident in children, but obesity may aggravate the effects of both TMAO and separate oxidative stress.

TMAO’s link to insulin resistance, glucose metabolism, and diabetes has been explored extensively, both in animal models (77, 128) and in human adults (34, 129–131). TMAO can impair glucose tolerance and induce adipose tissue inflammation in high-fat diet-fed mice (39). FMO3, which contributes to TMAO production, has been implicated in glucose and lipid metabolism impairment in mice and atherosclerosis (132). Reduced TMAO production through FMO3 inhibition can prevent hyperglycemia (80). Notably, circulating TMAO levels appear to correlate positively with fasting glucose levels in humans (133). The connection between TMAO and IR has been explored, but most studies are cross-sectional, making it challenging to determine causality. A longitudinal study reported elevated TMAO was associated with reduced diabetes risk (134). However, limited data exist on TMAO’s role in predicting type 2 diabetes development and its relation to early risk biomarkers like insulin resistance and prediabetes. One longitudinal study found a nonlinear association between TMAO levels and prediabetes prevalence, but no significant link to insulin resistance or fasting glucose change (135).

In the present study the obese subjects were divided into obese with IR, and without IR, according to HOMA-IR values. Although children with IR presented a greater weight excess (BMI, WC, WHR) and significantly higher vascular markers for atherosclerosis and arterial stiffness (CIMT, PWV, AIx, peripheral and central BP levels), serum TMAO levels did not differ significantly. No differences in sex, age, or puberty development were detected with regard to insulin resistance either. Serum TMAO did not correlate to HOMA-IR and insulin levels, but presented a negative moderate correlation to fasting glucose levels. Lower fasting glucose levels in insulin-resistant obese children might be explained by the fact that, unlike adults, children with pre-diabetic status present higher glucose variability, with highs and lows throughout a usual day (136). The multiple regression showed a significant influence of the blood parameters investigating IR (HOMA-IR, fasting glucose, VAI, and TyG index) on serum TMAO, but only HOMA-IR and fasting glucose were confirmed as independent predictors of TMAO. Moreover, when investigating the presence of acanthosis nigricans as a marker of insulin resistance (62), this study showed that an increase in TMAO is associated with an increase in the probability of the presence of acanthosis nigricans. Insulin resistance itself is an aggravating factor for the progression of vascular disruptions in obese children (137). Assessing the influence of HOMA-IR and other blood parameters (fasting glucose, lipid fractions, triglycerides, uric acid, creatinine, AST, ALT, 25-OH-Vitamin D), along with TMAO, on the vascular parameters showed interesting results. The multiple regression analysis showed that of all the blood parameters, only TMAO, uric acid, creatinine, and ALT are significant predictors of CIMT. After adding the blood tests into the model regression, TMAO, HOMA-IR, creatinine, and ALT remained predictors of PWV. As for the peripheral BP levels, TMAO, HOMA-IR, and creatinine remained predictors of SBP, while TMAO and ALT remained predictors of DBP. Although in the case of cSBP, TMAO, and HOMA-IR remained predictors after adding the other blood parameters in the model, for cDBP, only the HOMA-IR remained a significant predictor, while TMAO lost its influence. In the case of cPP, although TMAO did not keep its significance as a predictor while adding the clinical markers of adiposity, puberty, and age, it did not lose it after adding the other blood parameters: TMAO, HOMA-IR, fasting glucose, and the uric acid remain significant predictors. Neither TMAO nor the other blood parameters exert a significant influence on AIx. Hence, we can observe that both HOMA-IR and TMAO are significant predictors of the parameters measuring arterial stiffness, but only the TMAO of CIMT. This goes to show that a connection between IR and serum TMAO exists, but further research is needed to establish the causal-effect connections.

An unexpected finding of this study was the lack of connections between the lipid fractions and ratios and the TMAO levels and vascular markers we investigated, respectively. Contrary to our expectations, the controls presented a significantly lower HDL-c and higher TC/HDL-c ratio. The rest of the lipid fractions’ mean values were also higher in the control group, but not significantly so. We do not believe these results are in any way significant because, the lipid fractions of the controls, although being the opposite of what one would expect, did not present dyslipidemic values. A lower HDL-c in normal-weight children can be explained by sedentariness and increased consumption of saturated fats, characteristics that unfortunately can be applied to many children today, regardless of their BMI (138, 139). However, we must acknowledge the fact that in this study, the control group was quite small, and variations in blood tests mean values could be attributed to this fact also. A particularity of this studied obese group is that these participants did not present pathological lipid levels either. In our previous studies on obese children, we detected significantly lower HDLc levels that correlated negatively to CIMT values (16), higher LDL-c that correlated positively with CIMT and arterial stiffness markers, correlations between non-HDL and PWV, and triglycerides and PWV, BP levels, respectively (137). The associations between TMAO and lipid metabolism are mainly related to fatty acids and sugar intake, microbiota composition, and liver function (140). Several studies in adults have reported a connection between the makeup of gut bacteria and variations in blood lipid levels (141, 142). In animal models, metabolites like TMAO, carnitine, and γ-butyrobetaine have been shown to disrupt cholesterol metabolism (143–145). While one previous study discovered a negative association between plasma betaine and lipids such as LDL-c and triglycerides (146), there remains limited information regarding the links between TMAO-related metabolites and blood lipids. The results of our study suggest that the connections between TMAO and lipid metabolism are less significant than the connections to IR, but stress the importance of further investigations on larger study groups.

Although showing significantly lower values in obese children, 25-OH-Vitamin D did not present a significant correlation or influence on serum TMAO. Vitamin D deficiency represents almost a normality for cardio-metabolically impaired individuals, indifferently of their age (147, 148). A study on adults investigating the connections between the levels of vitamin D and TMAO, in relation to BMI and the presence of MAFLD, showed that low vitamin D levels (<19.83 ng/ml) are predictive of high serum TMAO levels, but a relation of causality was not identified (112).

The few studies investigating the role of TMAO in the pathogenesis of CV risk in women with PCOS have demonstrated higher levels of serum TMAO in comparison to same-age and BMI controls (149, 150). Moreover, one study associated these higher levels of TMAO with hyperandrogenism, as it showed that after a 3-months contraceptive administration and lifestyle intervention, the TMAO levels decreased significantly (149). The same team compared the microbiota of obese women with PCOS and of women with simple obesity and showed that those with PCOS presented a greater abundance of *Ruminococcaceae* (151), a genre associated with atherosclerosis and high TMAO levels in animal models (152). Hence, due to endocrine alterations (probably hyperandrogenism) and weight excess-related problems, women with PCOS seem to produce higher serum TMAO levels that may increase their CV risk (153). In this study, we looked at the cycle irregularity component of PCOS in the post-pubertal participants. Only 14 girls had completed their puberty, 50% of whom presented irregular menses. The ANOVA analysis showed that, although the obese girls with irregular menses presented a higher weight excess than the girls without menses and those with regular menstrual cycles, the TMAO levels among the three groups did not differ. We conclude that, given the results presented in adult women, further research is needed for obese adolescent girls associating PCOS, as our previous work has shown that these girls are likely to present increased arterial stiffness markers in the context of IR (138). Other studies have also connected the presence of PCOS to larger values of CIMT, even after adjusting for BMI, age, and smoking (154, 155). However, the central obesity of these individuals could also represent a significant factor in increasing arterial stiffness, hence correlations between PCOS and arterial damage in obese children are not definitive (156).

Recent research has emphasized the variations in microbiome composition between sexes, observed in both animal models and human studies, along with a reciprocal interaction between the microbiota and the endocrine system. More information regarding the connections between gut microbiota, gut-related metabolites, and sex hormones, in both children going through puberty and adults, is needed in order to draw conclusions. Both animal and human models have shown differences in gut microbiota composition between sexes: women host a higher ratio of the phyla *Firmicutes/Bacteroidetes* than men, a ratio that is significantly influenced by BMI, however; menopausal women and men present a higher abundance of the phyla *Bacteroidetes* and *Prevotella*. While sex itself may modify diet choices, the host’s sex hormones profile can also influence the composition of gut microbiota. Moreover, some bacteria have the capacity of producing, responding to, and regulate hormones. This is achieved by gene transcription inhibition and hormone conversion. Estradiol and testosterone may contribute to the sex-based differences in gut microbiota composition, potentially influencing the immune environment of the gut mucosa (157). Sex-related differences in FMO3 expression have been observed in mice, with consequent lower production of TMAO in male mice. These differences have been associated with testosterone levels, which may downregulate FMO3, explaining lower levels of TMAO in males compared to females (158). TMA, the substrate for TMAO, is higher in healthy men compared to healthy women. Lower TMA in men with cardiovascular disease has been reported in comparison to healthy men, while higher TMA was reported in cardiovascular female patients compared to healthy females (159). These variations could be linked to the redox and metabolic system because in normal conditions, females are less affected by oxidative stress which directly impacts cardiovascular health and atherosclerosis (160). This study did not detect significant differences in TMAO levels between sexes in the obese group or when comparing obese and controls according to sex. Normal-weight girls did present significantly lower TMAO levels compared to normal-weight boys and obese girls. Furthermore, the analysis did not link any differences in TMAO levels to puberty development. Further studies are needed in order to detect any connections between sex, the roles of gut microbiota, and TMAO, in pre-pubertal children, adolescents, and adults respectively, as differences between sexes have been clearly detected in adults in previous studies.

This study also revealed no differences between the TMAO levels of subjects in different stages of puberty development. Physiological changes during puberty of gut microbiota composition are cited in the literature, but the results are inconclusive. While some authors using conventional colony plating technology have cited no age differences (161), others, using DNA/RNA sequencing technology have shown clear differences both related to age (162) and puberty (163). TMA, TMAO, and other gut-derived metabolites should therefore be influenced by these changes, but studies are yet to prove that.

The key strength of this study is that it managed to confirm the connections between serum TMAO, childhood obesity, and early disruptive modifications of the arterial walls. The highlight of this work is that, to our knowledge, no other study has approached this subject as we have. Certain limitations should be considered, however. Firstly, the cross-sectional design prevents establishing a cause-and-effect relationship between TMAO, obesity, and vascular damage. Longitudinal studies are needed to determine the temporal sequence of these associations. Secondly, our control sample consisted of only 20 children, and this might be an impediment in interpreting the results. Lastly, the study did not investigate potential confounders, such as dietary habits, physical activity levels, and genetic predisposition, which could influence TMAO levels and vascular parameter dynamics. In the future, we plan to integrate these cofounders in our work and investigate the implications of liver health both in relation to TMAO and to vascular alterations. Another interesting approach would be to perform a longitudinal investigation and measure the effect of lifestyle intervention on both TMAO levels, and on the vascular parameters, to possibly show whether at young ages the vascular damage could be reversible.

In conclusion, our study provides compelling evidence supporting the link between serum TMAO, obesity, and vascular damage in children. The elevated TMAO levels observed in obese children suggest its potential as a biomarker for obesity. Moreover, the positive correlations between TMAO and subclinical atherosclerosis and arterial stiffness surrogate markers indicate the detrimental effects of TMAO on the vasculature. These findings highlight the importance of further research to unravel the underlying mechanisms and explore the potential of TMAO as a therapeutic target for obesity-related vascular complications. Understanding the relationship between TMAO, obesity, and cardiovascular risk may contribute to the development of personalized interventions aimed at reducing the burden of cardio-metabolic diseases in children and young adults struggling with obesity.

## Data availability statement

The raw data supporting the conclusions of this article will be made available by the authors, without undue reservation.

## Ethics statement

The studies involving humans were approved by The Ethics Committee for Scientific Research of the “Pius Brinzeu” Emergency County Hospital (No. 349/15.11.2022). The studies were conducted in accordance with the local legislation and institutional requirements. Written informed consent for participation in this study was provided by the participants’ legal guardians/next of kin.

## Author contributions

MM: Conceptualization, formal analysis, investigation, methodology, writing – original draft. CP: project administration, resources, validation, writing – review & editing. AB: Resources, software, visualization, writing – review & editing. CR: Software, visualization, writing – review & editing. DP: investigation, software, writing – review & editing. OV: Data curation, validation, writing – review & editing. IM: Formal analysis, methodology, project administration, writing – review & editing. DS: Conceptualization, funding acquisition, methodology, resources, supervision, validation, writing – review & editing.

## References

[1] DeBoer MD. Assessing and managing the metabolic syndrome in children and adolescents. Nutrients (2019) 11(8):1788. doi: 10.3390/nu11081788 31382417 PMC6723651

[2] Magnussen CG Koskinen J Chen W Thomson R Schmidt MD Srinivasan SR. Pediatric metabolic syndrome predicts adulthood metabolic syndrome, subclinical atherosclerosis, and type 2 diabetes mellitus but is no better than body mass index alone: The Bogalusa Heart Study and the Cardiovascular Risk in Young Finns Study. Circulation (2010) 122(16):1604–11. doi: 10.1161/CIRCULATIONAHA.110.940809 PMC3388503 20921439

[3] WHO European Regional Obesity Report 2022 (2022). Copenhagen: WHO Regional Office for Europe;. Available at: https://apps.who.int/iris/bitstream/handle/10665/353747/9789289057738-eng.pdf (Accessed 6 March 2023).

[4] Report on the fifth round of data collection, 2018–2020: WHO European Childhood Obesity Surveillance Initiative (COSI) (2022). Copenhagen: WHO Regional Office for Europe. Available at: file:///D:/Downloads/WHO-EURO-2022-6594-46360-67071-eng.pdf (Accessed 6 March 2023).

[5] Stierman B Afful J Carroll MD Chen TC Davy O. National Center for Health Statistics (U.S.). National Health and Nutrition Examination Survey 2017–March 2020 Prepandemic Data Files Development of Files and Prevalence Estimates for Selected Health Outcomes (2021). National Health Statistics Reports. Available at: https://stacks.cdc.gov/view/cdc/106273 (Accessed 6 March 2023). 10.15620/cdc:106273 PMC11513744 39380201

[6] Lange SJ Kompaniyets L Freedman DS Kraus EM Porter R Blanck HM. Longitudinal trends in body mass index before and during the COVID-19 pandemic among persons aged 2–19 years — United states, 2018–2020. MMWR Morb Mortal Wkly Rep (2021) 70(37):1278–83. doi: 10.15585/mmwr.mm7037a3 PMC8445379 34529635

[7] Woolford SJ Sidell M Li X Else V Young DR Resnicow K. Changes in body mass index among children and adolescents during the COVID-19 pandemic. JAMA (2021) 326(14):1434–6. doi: 10.1001/jama.2021.15036 PMC8511973 34448817

[8] Balasundaram P Krishna S. Obesity effects on child health, in: StatPearls (2022). Treasure Island (FL: StatPearls Publishing. Available at: https://www.ncbi.nlm.nih.gov/books/NBK570613/ (Accessed 6 March 2023). 34033375

[9] Zieske AW Malcom GT Strong JP. Natural history and risk factors of atherosclerosis in children and youth: The PDAY study. Pediatr Pathol Mol Med (2002) 21(3):213–37. doi: 10.1080/15227950252852104 11942537

[10] Abraham TM Pedley A Massaro JM Hoffmann U Fox CS. Association between visceral and subcutaneous adipose depots and incident cardiovascular disease risk factors. Circulation (2015) 132(17):1639–47. doi: 10.1161/CIRCULATIONAHA.114.015000 PMC4779497 26294660

[11] Liu Y Dai M. Trimethylamine N-oxide generated by the gut microbiota is associated with vascular inflammation: new insights into atherosclerosis. Mediators Inflamm (2020) 2020:4634172. doi: 10.1155/2020/4634172 32148438 PMC7048942

[12] Chen K Zheng X Feng M Li D Zhang H. Gut microbiota-dependent metabolite trimethylamine N-oxide contributes to cardiac dysfunction in western diet-induced obese mice. Front Physiol (2017) 8:139. doi: 10.3389/fphys.2017.00139 28377725 PMC5359299

[13] Kanitsoraphan C Rattanawong P Charoensri S Senthong V. Trimethylamine N-oxide and risk of cardiovascular disease and mortality. Curr Nutr Rep (2018) 7(4):207–13. doi: 10.1007/s13668-018-0252-z 30362023

[14] Li XS Obeid S Wang Z Hazen BJ Li L Wu Y. Trimethyl lysine, a trimethylamine N-oxide precursor, provides near- and long-term prognostic value in patients presenting with acute coronary syndromes. Eur Heart J (2019) 40(32):2700–9. doi: 10.1093/eurheartj/ehz259 PMC7963132 31049589

[15] Wang Z Tang WH Buffa JA Fu X Britt EB Koeth RA. Prognostic value of choline and betaine depends on intestinal microbiota-generated metabolite trimethylamine-N-oxide. Eur Heart J (2014) 35(14):904–10. doi: 10.1093/eurheartj/ehu002 PMC3977137 24497336

[16] Mihuta M-S Paul C Ciulpan A Dacca F Velea IP Mozos I. Subclinical atherosclerosis progression in obese children with relevant cardiometabolic risk factors can be assessed through carotid intima-media thickness. Appl Sci (2021) 11(22):10721. doi: 10.3390/app112210721

[17] Yasin J Sharma C Hashim MJ Al Hamed S AlKaabi J Aburawi EH. Cross-sectional association between body fat composition and biomarkers of inflammation and endothelial dysfunction in children with overweight/obesity. Diabetes Metab Syndr Obes (2023) 16:483–93. doi: 10.2147/DMSO.S390071 PMC9942511 36824321

[18] Yonemori KM Lim U Koga KR Wilkens LR Au D Boushey CJ. Dietary choline and betaine intakes vary in an adult multiethnic population. J Nutr (2013) 143(6):894–9. doi: 10.3945/jn.112.171132 PMC3652885 23616508

[19] Yu ZL Zhang LY Jiang XM Xue CH Chi N Zhang TT. Effects of dietary choline, betaine, and L-carnitine on the generation of trimethylamine-N-oxide in healthy mice. J Food Sci (2020) 85(7):2207–15. doi: 10.1111/1750-3841.15186 32572979

[20] Zhang AQ Mitchell SC Smith RL. Dietary precursors of trimethylamine in man: A pilot study. Food Chem Toxicol (1999) 37(5):515–20. doi: 10.1016/S0278-6915(99)00028-9 10456680

[21] Zeisel SH Warrier M. TrimethylamineN-oxide, the microbiome, and heart and kidney disease. Annu Rev Nutr (2017) 37:157–81. doi: 10.1146/annurev-nutr-071816-064732 28715991

[22] Li X Hong J Wang Y Pei M Wang L Gong Z. Trimethylamine-N-oxide pathway: A potential target for the treatment of MAFLD. Front Mol Biosci (2021) 8:733507. doi: 10.3389/fmolb.2021.733507 34660695 PMC8517136

[23] Fu BC Hullar MAJ Randolph TW Franke AA Monroe KR Cheng I. Associations of plasma trimethylamine N-oxide, choline, carnitine, and betaine with inflammatory and cardiometabolic risk biomarkers and the fecal microbiome in the multiethnic cohort adiposity phenotype study. Am J Clin Nutr (2020) 111(6):1226–34. doi: 10.1093/ajcn/nqaa015 PMC7266689 32055828

[24] Qiu L Tao X Xiong H Yu J Wei H. Lactobacillus plantarumZDY04 exhibits a strain-specific property of lowering TMAOviathe modulation of gut microbiota in mice. Food Funct (2018) 9(8):4299–309. doi: 10.1039/c8fo00349a 30039147

[25] Cantero MA Guedes MRA Fernandes R Lollo PCB. Trimethylamine N-oxide reduction is related to probiotic strain specificity: A systematic review. Nutr Res (2022) 104:29–35. doi: 10.1016/j.nutres.2022.04.001 35588611

[26] Turnbaugh PJ Hamady M Yatsunenko T Cantarel BL Duncan A Ley RE. A core gut microbiome in obese and lean twins. Nature (2009) 457(7228):480–4. doi: 10.1038/nature07540 PMC2677729 19043404

[27] Turnbaugh PJ Ley RE Mahowald MA Magrini V Mardis ER Gordon JI. An obesity-associated gut microbiome with increased capacity for energy harvest. Nature (2006) 444(7122):1027–31. doi: 10.1038/nature05414 17183312

[28] Fetissov SO. Role of the gut microbiota in host appetite control: bacterial growth to animal feeding behavior. Nat Rev Endocrinol (2017) 13(1):11–25. doi: 10.1038/nrendo.2016.150 27616451

[29] Musso G Gambino R Cassader M. Obesity, diabetes, and gut microbiota: the hygiene hypothesis expanded? Diabetes Care (2010) 33(10):2277–84. doi: 10.2337/dc10-0556 PMC2945175 20876708

[30] Backhed F Manchester JK Semenkovich CF Gordon JI. Mechanisms underlying the resistance to diet-induced obesity in germ-free mice. Proc Natl Acad Sci USA (2007) 104(3):979–84. doi: 10.1073/pnas.0605374104 PMC1764762 17210919

[31] Ley RE Turnbaugh PJ Klein S Gordon JI. Microbial ecology: human gut microbes associated with obesity. Nature (2006) 444(7122):1022–3. doi: 10.1038/4441022a 17183309

[32] Ley RE Backhed F Turnbaugh P Lozupone CA Knight RD Gordon JI. Obesity alters gut microbial ecology. Proc Natl Acad Sci USA (2005) 102(31):11070–5. doi: 10.1073/pnas.0504978102 PMC1176910 16033867

[33] Backhed F Ding H Wang T Hooper LV Koh GY Nagy A. The gut microbiota as an environmental factor that regulates fat storage. Proc Natl Acad Sci USA (2004) 101(44):15718–23. doi: 10.1073/pnas.0407076101 PMC524219 15505215

[34] Dambrova M Latkovskis G Kuka J Strele I Konrade I Grinberga S. Diabetes is associated with higher trimethylamine N-oxide plasma levels. Exp Clin Endocrinol Diabetes (2016) 124(4):251–6. doi: 10.1055/s-0035-1569330 27123785

[35] Dong F Jiang S Tang C Wang X Ren X Wei Q. Trimethylamine N-oxide promotes hyperoxaluria-induced calcium oxalate deposition and kidney injury by activating autophagy. Free Radic Biol Med (2021) 179:288–300. doi: 10.1016/j.freeradbiomed.2021.11.010 34767921

[36] Xu R Wang Q Li L. A genome-wide systems analysis reveals strong link between colorectal cancer and trimethylamine N-oxide (TMAO), a gut microbial metabolite of dietary meat and fat. BMC Genomics (2015) 16(Suppl 7):S4. doi: 10.1186/1471-2164-16-S7-S4 PMC4474417 26100814

[37] Baker JR Struempler A Chaykin S. A comparative study of trimethylamine-N-oxide biosynthesis. Biochim Biophys Acta (1963) 71:58–64. doi: 10.1016/0006-3002(63)90985-5 13969156

[38] Paul B Barnes S Demark-Wahnefried W Morrow C Salvador C Skibola C. Influences of diet and the gut microbiome on epigenetic modulation in cancer and other diseases. Clin Epigenet (2015) 7:112. doi: 10.1186/s13148-015-0144-7 PMC4609101 26478753

[39] Gao X Liu X Xu J Xue C Xue Y Wang Y. Dietary trimethylamine N-oxide exacerbates impaired glucose tolerance in mice fed a high fat diet. J Biosci Bioeng (2014) 118(4):476–81. doi: 10.1016/j.jbiosc.2014.03.001 24721123

[40] Tong M Neusner A Longato L Lawton M Wands JR de la Monte SM. Nitrosamine exposure causes insulin resistance diseases: relevance to type 2 diabetes mellitus, non-alcoholic steatohepatitis, and Alzheimer's disease. J Alzheimers Dis (2009) 17(4):827–44. doi: 10.3233/JAD-2009-1115 PMC2952429 20387270

[41] de la Monte SM Tong M Lawton M Longato L. Nitrosamine exposure exacerbates high fat diet-mediated type 2 diabetes mellitus, non-alcoholic steatohepatitis, and neurodegeneration with cognitive impairment. Mol Neurodegener (2009) 4:54. doi: 10.1186/1750-1326-4-54 20034403 PMC2803782

[42] Chen YM Liu Y Zhou RF Chen XL Wang C Tan XY. Associations of gut-flora-dependent metabolite trimethylamine-N-oxide, betaine and choline with non-alcoholic fatty liver disease in adults. Sci Rep (2016) 6:19076. doi: 10.1038/srep19076 26743949 PMC4705470

[43] Stols-Gonçalves D Hovingh GK Nieuwdorp M Holleboom AG. NAFLD and atherosclerosis: two sides of the same dysmetabolic coin? Trends Endocrinol Metab (2019) 30(12):891–902. doi: 10.1016/j.tem.2019.08.008 31630897

[44] Pierce GL Roy SJ Gimblet CJ. The gut-arterial stiffness axis: is TMAO a novel target to prevent age-related aortic stiffening? Hypertens (2021) 78(2):512–5. doi: 10.1161/HYPERTENSIONAHA.121.17487 PMC8274311 34232682

[45] Powell-Wiley TM Poirier P Burke LE Després JP Gordon-Larsen P Lavie CJ. Obesity and cardiovascular disease: A scientific statement from the american heart association. Circulation (2021) 143:e984–e1010. doi: 10.1161/CIR.0000000000000973 33882682 PMC8493650

[46] Tounian P Aggoun Y Dubern B Varille V Guy-Grand B Sidi D. Presence of increased stiffness of the common carotid artery and endothelial dysfunction in severely obese children: A prospective study. Lancet (2001) 358:1400–4. doi: 10.1016/S0140-6736(01)06525-4 11705484

[47] Bots ML Hofman A De Jong PT Grobbee DE. Common carotid intima-media thickness as an indicator of atherosclerosis at other sites of the carotid artery. Rotterdam Study Ann Epidemiol (1996) 6(2):147–53. doi: 10.1016/1047-2797(96)00001-4 8775595

[48] Beauloye V Zech F Tran HT Clapuyt P Maes M Brichard SM. Determinants of early atherosclerosis in obese children and adolescents. J Clin Endocrinol Metab (2007) 92:3025–32. doi: 10.1210/jc.2007-0619 17519311

[49] Farello G Antenucci A Stagi S Mazzocchetti C Ciocca F Verrotti A. Metabolically healthy and metabolically unhealthy obese children both have increased carotid intima-media thickness: A case control study. BMC Cardiovasc Disord (2018) 18:1–6. doi: 10.1186/s12872-018-0874-5 29973145 PMC6032770

[50] Mihuta MS Stoian D Borlea A Roi CM Velea-Barta OA Mozos I. Evaluating the arterial stiffness as a useful tool in the management of obese children. Children (2023) 10:183. doi: 10.3390/children10020183 36832311 PMC9955158

[51] Reusz GS Cseprekal O Temmar M Kis E Cherif AB Thaleb A. Reference values of pulse wave velocity in healthy children and teenagers. Hypertens (2010) 56:217–24. doi: 10.1161/HYPERTENSIONAHA.110.152686 20566959

[52] Mozos I Maidana JP Stoian D Stehlik M. Gender differences of arterial stiffness and arterial age in smokers. Int J Environ Res Public Health (2017) 14:565. doi: 10.3390/ijerph14060565 28587127 PMC5486251

[53] Berenson GS Srinivasan SR Bao W 3rd NWP Tracy RE Wattigney WA. Association between multiple cardiovascular risk factors and atherosclerosis in children and young adults. The Bogalusa Heart Study. N Engl J Med (1998) 338:1650–6. doi: 10.1056/NEJM199806043382302 9614255

[54] Balasundaram P Krishna S. Obesity effects on child health, in: StatPearls (2022). Treasure Island, FL, USA: StatPearls Publishing. Available at: https://www.ncbi.nlm.nih.gov/books/NBK570613/ (Accessed July 1st 2023). 34033375

[55] Xi B Zong X Kelishadi R Litwin M Hong YM Poh BK. International waist circumference percentile cutoffs for central obesity in children and adolescents aged 6 to 18 years. J Clin Endocrinol Metab (2020) 105(4):e1569–83. doi: 10.1210/clinem/dgz195 PMC7059990 31723976

[56] Cheung YF. Arterial stiffness in the young: Assessment, determinants, and implications. Korean Circ J (2010) 40:153–62. doi: 10.4070/kcj.2010.40.4.153 PMC2859331 20421954

[57] Velea IP Albulescu R Arghirescu ST. Obezitatea la copil. In: Velea I, editor. Pediatrie-Curs Pentru Studentii Facultătii de Medicină: Obezitatea la Copil. Timisoara, Romania: Editura Victor Babes (2016). p. 289–97.

[58] Hsu CN Chang-Chien GP Lin S Hou CY Lu PC Tain YL. Association of trimethylamine, trimethylamine N-oxide, and dimethylamine with cardiovascular risk in children with chronic kidney disease. J Clin Med (2020) 9(2):336. doi: 10.3390/jcm9020336 31991725 PMC7074377

[59] Hampl SE Hassink SG Skinner AC Armstrong SC Barlow SE Bolling CF. Clinical practice guideline for the evaluation and treatment of children and adolescents with obesity. Pediatrics (2023) 151(2):e2022060640. doi: 10.1542/peds.2022-060640 36622115

[60] Santoro N Amato A Grandone A Brienza C Savarese P Tartaglione N. Predicting metabolic syndrome in obese children and adolescents: look, measure and ask. Obes Facts (2013) 6:48–56. doi: 10.1159/000348625 23429241 PMC5644669

[61] Emmanuel M Bokor BR. Tanner stages, in: StatPearls (2022). Treasure Island (FL: StatPearls Publishing. Available at: https://www.ncbi.nlm.nih.gov/books/NBK470280/ (Accessed January 7, 2023). 29262142

[62] Radu AM Carsote M Dumitrascu MC Sandru F. Acanthosis nigricans: pointer of endocrine entities. Diagnostics (Basel) (2022) 12(10):2519. doi: 10.3390/diagnostics12102519 36292208 PMC9600076

[63] Matthews DR Hosker JP Rudenski AS Naylor BA Treacher DF Turner RC. Homeostasis model assessment: insulin resistance and beta-cell function from fasting plasma glucose and insulin concentrations in man. Diabetologia (1985) 28:412–9. doi: 10.1007/BF00280883 3899825

[64] Vizzuso S Del Torto A Dilillo D Calcaterra V Di Profio E Leone A. Visceral Adiposity Index (VAI) in children and adolescents with obesity: no association with daily energy intake but promising tool to identify Metabolic Syndrome (MetS). Nutrients (2021) 13(2):413. doi: 10.3390/nu13020413 33525454 PMC7911630

[65] Shashaj B Luciano R Contoli B Morino GS Spreghini MR Rustico C. Reference ranges of HOMA-IR in normal-weight and obese young Caucasians. Acta Diabetol (2016) 53:251–60. doi: 10.1007/s00592-015-0782-4 26070771

[66] Amato MC Giordano C Galia M Criscimanna A Vitabile S Midiri M. Visceral Adiposity Index: a reliable indicator of visceral fat function associated with cardiometabolic risk. Diabetes Care (2010) 33(4):920–2. doi: 10.2337/dc09-1825 PMC2845052 20067971

[67] Calcaterra V Montalbano C de Silvestri A Pelizzo G Regalbuto C Paganelli V. Triglyceride glucose index as a surrogate measure of insulin sensitivity in a caucasian pediatric population. J Clin Res Pediatr Endocrinol (2019) 1:1–11. doi: 10.4274/jcrpe.galenos.2019.2019.0024 31088046

[68] Polak JF Meisner A Pencina MJ Wolf PA D'Agostino RB. Variations in common carotid artery intima-media thickness during the cardiac cycle: implications for cardiovascular risk assessment. J Am Soc Echocardiogr (2012) 25(9):1023–8. doi: 10.1016/j.echo.2012.05.007 PMC3544292 22721828

[69] Gómez-Marcos MA Recio-Rodríguez JI Patino-Alonso MC Agudo-Conde C Gómez-Sanchez L Gómez-Sanchez M. Protocol for measuring carotid intima-media thickness that best correlates with cardiovascular risk and target organ damage. Am J Hypertens (2012) 25:955–61. doi: 10.1038/ajh.2012.72 22717546

[70] El Jalbout R. Intima-media thickness measurement in children; techniques and reference values. Am J BioMed Sci Res (2020) 7(2):101–3. doi: 10.34297/AJBSR.2020.07.001123

[71] Freire CM Ribeiro AL Barbosa FB Lana AM e Silva AC Ribeiro-Oliveira A. Comparison between automated and manual measurements of carotid intima-media thickness in clinical practice. Vasc Health Risk Manag (2009) 5:811–7. doi: 10.2147/VHRM.S5745 PMC2754094 19812693

[72] Vermeersch S Rietzschel E De Buyzere M Van Bortel LM D’Asseler Y Gillebert TC. Validation of a new automated IMT measurement algorithm. J Hum Hypertens (2007) 21:976–8. doi: 10.1038/sj.jhh.100225 17568751

[73] Mihuta MS Paul C Borlea A Cepeha CM Velea IP Mozos I. The oscillometric pulse wave analysis is useful in evaluating the arterial stiffness of obese children with relevant cardiometabolic risks. J Clin Med (2022) 11:5078. doi: 10.3390/jcm11175078 36079009 PMC9457050

[74] Papandreou C Moré M Bellamine A. Trimethylamine N-oxide in relation to cardiometabolic health—Cause or effect? Nutrients (2020) 12:1330. doi: 10.3390/nu12051330 32392758 PMC7284902

[75] Lindskog Jonsson A Caesar R Akrami R Reinhardt C Fåk Hållenius F Borén J. Impact of gut microbiota and diet on the development of atherosclerosis in apoe-/- mice. Arterioscler Thromb Vasc Biol (2018) 38(10):2318–26. doi: 10.1161/ATVBAHA.118.311233 PMC6166703 29903735

[76] Zhao ZH Xin FZ Zhou D Xue YQ Liu XL Yang RX. Trimethylamine N-oxide attenuates high-fat high-cholesterol diet-induced steatohepatitis by reducing hepatic cholesterol overload in rats. World J Gastroenterol (2019) 25(20):2450–62. doi: 10.3748/wjg.v25.i20.2450 PMC6543245 31171889

[77] Lanz M Janeiro MH Milagro FI Puerta E Ludwig IA Pineda-Lucena A. Trimethylamine N-oxide (TMAO) drives insulin resistance and cognitive deficiencies in a senescence accelerated mouse model. Mech Ageing Dev (2022) 204:111668. doi: 10.1016/j.mad.2022.111668 35341897

[78] Zhang W Miikeda A Zuckerman J Jia X Charugundla S Zhou Z. Inhibition of microbiota-dependent TMAO production attenuates chronic kidney disease in mice. Sci Rep (2021) 11(1):518. doi: 10.1038/s41598-020-80063-0 33436815 PMC7804188

[79] Li C Zhu L Dai Y Zhang Z Huang L Wang TJ. Diet-induced high serum levels of trimethylamine-N-oxide enhance the cellular inflammatory response without exacerbating acute intracerebral hemorrhage injury in mice. Oxid Med Cell Longev (2022) 2022:1599747. doi: 10.1155/2022/1599747 35242275 PMC8886754

[80] Miao J Ling AV Manthena PV Gearing ME Graham MJ Crooke RM. Flavin-containing monooxygenase 3 as a potential player in diabetes-associated atherosclerosis. Nat Commun (2015) 6:6498. doi: 10.1038/ncomms7498 25849138 PMC4391288

[81] Chen S Henderson A Petriello MC Romano KA Gearing M Miao J. Trimethylamine N-oxide binds and activates PERK to promote metabolic dysfunction. Cell Metab (2019) 30(6):1141–1151.e5. doi: 10.1016/j.cmet.2019.08.021 31543404

[82] Duttaroy AK. Role of gut microbiota and their metabolites on atherosclerosis, hypertension and human blood platelet function: A review. Nutrients (2021) 13(1):144. doi: 10.3390/nu13010144 33401598 PMC7824497

[83] Heianza Y Sun D Li X DiDonato JA Bray GA Sacks FM. Gut microbiota metabolites, amino acid metabolites and improvements in insulin sensitivity and glucose metabolism: the POUNDS Lost trial. Gut (2019) 68(2):263–70. doi: 10.1136/gutjnl-2018-316155 PMC6275143 29860242

[84] DiNicolantonio JJ McCarty M OKeefe J. Association of moderately elevated trimethylamine N-oxide with cardiovascular risk: is TMAO serving as a marker for hepatic insulin resistance. Open Heart (2019) 6:e000890. doi: 10.1136/openhrt-2018-000890 30997120 PMC6443140

[85] Zhu T Goodarzi MO. Metabolites linking the gut microbiome with risk for type 2 diabetes. Curr Nutr Rep (2020) 9(2):83–93. doi: 10.1007/s13668-020-00307-3 32157661 PMC7282969

[86] Eyileten C Jarosz-Popek J Jakubik D Gasecka A Wolska M Ufnal M. Plasma trimethylamine-N-oxide is an independent predictor of long-term cardiovascular mortality in patients undergoing percutaneous coronary intervention for acute coronary syndrome. Front Cardiovasc Med (2021) 8:728724. doi: 10.3389/fcvm.2021.728724 34778397 PMC8585769

[87] Yu D Shu X Rivera ES Zhang X Cai Q Calcutt MW. Urinary levels of trimethylamine-N-oxide and incident coronary heart disease: A prospective investigation among urban chinese adults. J Am Heart Assoc (2019) 8(2):e010606. doi: 10.1161/JAHA.118.010606 30606084 PMC6405718

[88] Xu J Cheng A Song B Zhao M Xue J Wang A. Trimethylamine N-oxide and stroke recurrence depends on ischemic stroke subtypes. Stroke (2022) 53(4):1207–15. doi: 10.1161/STROKEAHA.120.031443 34794334

[89] Nam HS. Gut microbiota and ischemic stroke: the role of trimethylamine N-oxide. J Stroke (2019) 21(2):151–9. doi: 10.5853/jos.2019.00472 PMC6549071 31161760

[90] Tang WH Wang Z Kennedy DJ Wu Y Buffa JA Agatisa-Boyle B. Gut microbiota-dependent trimethylamine N-oxide (TMAO) pathway contributes to both development of renal insufficiency and mortality risk in chronic kidney disease. Circ Res (2015) 116(3):448–55. doi: 10.1161/CIRCRESAHA.116.305360 PMC4312512 25599331

[91] Stubbs JR House JA Ocque AJ Zhang S Johnson C Kimber C. Serum trimethylamine-N-oxide is elevated in CKD and correlates with coronary atherosclerosis burden. J Am Soc Nephrol (2016) 27(1):305–13. doi: 10.1681/ASN.2014111063 PMC4696571 26229137

[92] Schiattarella GG Sannino A Toscano E Giugliano G Gargiulo G Franzone A. Gut microbe-generated metabolite trimethylamine-N-oxide as cardiovascular risk biomarker: A systematic review and dose-response meta-analysis. Eur Heart J (2017) 38(39):2948–56. doi: 10.1093/eurheartj/ehx342 29020409

[93] He S Jiang H Zhuo C Jiang W. Trimethylamine/trimethylamine-N-oxide as a key between diet and cardiovascular diseases. Cardiovasc Toxicol (2021) 21(6):593–604. doi: 10.1007/s12012-021-09656-z 34003426

[94] Kuo CH Liu CH Wang JH Hsu BG. Serum trimethylamine N-oxide levels correlate with metabolic syndrome in coronary artery disease patients. Int J Environ Res Public Health (2022) 19(14):8710. doi: 10.3390/ijerph19148710 35886563 PMC9318326

[95] Dumas ME Kinross J Nicholson JK. Metabolic phenotyping and systems biology approaches to understanding metabolic syndrome and fatty liver disease. Gastroenterology (2014) 146(1):46–62. doi: 10.1053/j.gastro.2013.11.001 24211299

[96] Ji Y Yin Y Sun L Zhang W. The molecular and mechanistic insights based on gut-liver axis: nutritional target for non-alcoholic fatty liver disease (NAFLD) improvement. Int J Mol Sci (2020) 21(9):3066. doi: 10.3390/ijms21093066 32357561 PMC7247681

[97] Theofilis P Vordoni A Kalaitzidis RG. Trimethylamine N-oxide levels in non-alcoholic fatty liver disease: A systematic review and meta-analysis. Metabolites (2022) 12(12):1243. doi: 10.3390/metabo12121243 36557281 PMC9784457

[98] Polak K Bergler-Czop B Szczepanek M Wojciechowska K Frątczak A Kiss N. Psoriasis and gut microbiome—Current state of art. Int J Mol Sci (2021) 22(9):4529. doi: 10.3390/ijms22094529 33926088 PMC8123672

[99] Oellgaard J Winther SA Hansen TS Rossing P von Scholten BJ. Trimethylamine N-oxide (TMAO) as a new potential therapeutic target for insulin resistance and cancer. Curr Pharm Des (2017) 23(25):3699–712. doi: 10.2174/1381612823666170622095324 28641532

[100] Buawangpong N Pinyopornpanish K Siri-Angkul N Chattipakorn N Chattipakorn SC. The role of trimethylamine-N-Oxide in the development of Alzheimer’s disease. J Cell Physiol (2022) 237(3):1661–85. doi: 10.1002/jcp.30646 34812510

[101] Dai Y Zhang J Wang Z Xu S Zhang Q Duan Z. Associations of diet with urinary trimethylamine-N-oxide (TMAO) and its precursors among free-living 10-year-old children: data from SMBCS. Nutrients (2022) 14(16):3419. doi: 10.3390/nu14163419 36014922 PMC9413070

[102] Iannotti LL Lutter CK Waters WF Gallegos Riofrío CA Malo C Reinhart G. Eggs early in complementary feeding increase choline pathway biomarkers and DHA: a randomized controlled trial in Ecuador. Am J Clin Nutr (2017) 106(6):1482–9. doi: 10.3945/ajcn.117.160515 PMC5698841 29092879

[103] Andraos S Lange K Clifford SA Jones B Thorstensen EB Kerr JA. Plasma trimethylamine N-oxide (TMAO) and its precursors: population epidemiology, parent-child concordance, and associations with reported dietary intake in 11–12-year-old children and their parents. Curr Dev Nutr (2020) 4(7):nzaa103. doi: 10.1093/cdn/nzaa103 32666035 PMC7335361

[104] Noce A Marchetti M Marrone G Di Renzo L Di Lauro M Di Daniele F. Link between gut microbiota dysbiosis and chronic kidney disease. Eur Rev Med Pharmacol Sci (2022) 26(6):2057–74. doi: 10.26355/eurrev_202203_28354 35363356

[105] Hsu CN Lu PC Lo MH Lin IC Chang-Chien GP Lin S. Gut microbiota-dependent trimethylamine N-oxide pathway associated with cardiovascular risk in children with early-stage chronic kidney disease. Int J Mol Sci (2018) 19(12):3699. doi: 10.3390/ijms19123699 30469463 PMC6320870

[106] Quan L Yi J Zhao Y Zhang F Shi XT Feng Z. Plasma trimethylamine N-oxide, a gut microbe-generated phosphatidylcholine metabolite, is associated with autism spectrum disorders. Neurotoxicology (2020) 76:93–8. doi: 10.1016/j.neuro.2019.10.012 PMC7385710 31704102

[107] Mu C Corley MJ Lee RWY Wong M Pang A Arakaki G. Metabolic framework for the improvement of autism spectrum disorders by a modified ketogenic diet: A pilot study. J Proteome Res (2019) 19:382–90. doi: 10.1021/acs.jproteome.9b00581 31696714

[108] Abrahamsson TR Jakobsson HE Andersson AF Bjorksten B Engstrand L Jenmalm MC. Low gut microbiota diversity in early infancy precedes asthma at school age. Clin Exp Allergy (2014) 44:842–50. doi: 10.1111/cea.12253 24330256

[109] Chiu C-Y Cheng M-L Chiang M-H Wang C-J Tsai M-H Lin G. Metabolomic analysis reveals distinct profiles in the plasma and urine associated with IgE reactions in childhood asthma. J Clin Med (2020) 9:887. doi: 10.3390/jcm9030887 32213896 PMC7141511

[110] Stroup BM Nair N Murali SG Broniowska K Rohr F Levy HL. Metabolomic markers of essential fatty acids, carnitine, and cholesterol metabolism in adults and adolescents with phenylketonuria. J Nutr (2018) 148(2):194–201. doi: 10.1093/jn/nxx039 29490096 PMC6251508

[111] Leal-Witt MJ Llobet M Samino S Castellano P Cuadras D Jimenez-Chillaron JC. Lifestyle intervention decreases urine trimethylamine N-oxide levels in prepubertal children with obesity. Obes (Silver Spring) (2018) 26(10):1603–10. doi: 10.1002/oby.22271 30204940

[112] Barrea L Muscogiuri G Annunziata G Laudisio D de Alteriis G Tenore GC. A new light on vitamin D in obesity: A novel association with trimethylamine-N-Oxide (TMAO). Nutrients (2019) 11(6):1310. doi: 10.3390/nu11061310 31185686 PMC6627576

[113] Dubinski P Czarzasta K Cudnoch-Jedrzejewska A. The influence of gut microbiota on the cardiovascular system under conditions of obesity and chronic stress. Curr Hypertens Rep (2021) 23(5):31. doi: 10.1007/s11906-021-01144-7 34014393 PMC8137478

[114] Gupta A Osadchiy V Mayer EA. Brain-gut-microbiome interactions in obesity and food addiction. Nat Rev Gastroenterol Hepatol (2020) 17(11):655–72. doi: 10.1038/s41575-020-0341-5 PMC7841622 32855515

[115] Cho I Yamanishi S Cox L Methe BA Zavadil J Li K. Antibiotics in early life alter the murine colonic microbiome and adiposity. Nature (2012) 488:621–6. doi: 10.1038/nature11400 PMC3553221 22914093

[116] Nobel YR Cox LM Kirigin FF Bokulich NA Yamanishi S Teitler I. Metabolic and metagenomic outcomes from early-life pulsed antibiotic treatment. Nat Commun (2015) 6:7486. doi: 10.1038/ncomms8486 26123276 PMC4491183

[117] Ruiz VE Battaglia T Kurtz ZD Bijnens L Ou A Engstrand I. A single early-in-life macrolide course has lasting effects on murine microbial network topology and immunity. Nat Commun (2017) 8:518. doi: 10.1038/s41467-017-00531-6 28894149 PMC5593929

[118] Zhang XS Li J Krautkramer KA Badri M Battaglia T Borbet TC. Antibiotic-induced acceleration of type 1 diabetes alters maturation of innate intestinal immunity. Elife (2018) 7:e37816. doi: 10.7554/eLife.37816 30039798 PMC6085123

[119] Livanos AE Greiner TU Vangay P Pathmasiri W Stewart D McRitchie S. Antibiotic-mediated gut microbiome perturbation accelerates development of type 1 diabetes in mice. Nat Microbiol (2016) 1:16140. doi: 10.1038/nmicrobiol.2016.140 27782139 PMC5808443

[120] Zhang C Yin A Li H Wang R Wu G Shen J. Dietary modulation of gut microbiota contributes to alleviation of both genetic and simple obesity in children. EBioMedicine (2015) 2(8):968–84. doi: 10.1016/j.ebiom.2015.07.007 PMC4563136 26425705

[121] Andraos S Jones B Lange K Clifford SA Thorstensen EB Kerr JA. Trimethylamine N-oxide (TMAO) is not associated with cardiometabolic phenotypes and inflammatory markers in children and adults. Curr Dev Nutr (2020) 5(1):nzaa179. doi: 10.1093/cdn/nzaa179 33501405 PMC7813154

[122] Randrianarisoa E Lehn-Stefan A Wang X Hoene M Peter A Heinzmann SS. Relationship of serum trimethylamine N-oxide (TMAO) levels with early atherosclerosis in humans. Sci Rep (2016) 6:26745. doi: 10.1038/srep26745 27228955 PMC4882652

[123] Brunt VE Casso AG Gioscia-Ryan RA Sapinsley ZJ Ziemba BP Clayton ZS. Gut microbiome-derived metabolite trimethylamine N-oxide induces aortic stiffening and increases systolic blood pressure with aging in mice and humans. Hypertension (2021) 78(2):499–511. doi: 10.1161/HYPERTENSIONAHA.120.16895 33966451 PMC8266738

[124] Brunt VE LaRocca TJ Bazzoni AE Sapinsley ZJ Miyamoto-Ditmon J Gioscia-Ryan RA. The gut microbiome-derived metabolite trimethylamine N-oxide modulates neuroinflammation and cognitive function with aging. Geroscience (2021) 43:377–94. doi: 10.1007/s11357-020-00257-2 PMC8050157 32862276

[125] Wang Z Levison BS Hazen JE Donahue L Li XM Hazen SL. Measurement of trimethylamine-N-oxide by stable isotope dilution liquid chromatography tandem mass spectrometry. Anal Biochem (2014) 455:35–40. doi: 10.1016/j.ab.2014.03.016 24704102 PMC4167037

[126] Brunt VE Gioscia-Ryan RA Casso AG VanDongen NS Ziemba BP Sapinsley ZJ. Trimethylamine-N-oxide promotes age-related vascular oxidative stress and endothelial dysfunction in mice and healthy humans. Hypertension (2020) 76:101–12. doi: 10.1161/HYPERTENSIONAHA.120.14759 PMC7295014 32520619

[127] Brunt VE Gioscia-Ryan RA Richey JJ Zigler MC Cuevas LM Gonzalez A. Suppression of the gut microbiome ameliorates age-related arterial dysfunction and oxidative stress in mice. J Physiol (2019) 597(9):2361–78. doi: 10.1113/JP277336 PMC6487935 30714619

[128] Gao X Du L Randell E Zhang H Li K Li D. Effect of different phosphatidylcholines on high fat diet-induced insulin resistance in mice. Food Funct (2021) 12(4):1516–28. doi: 10.1039/d0fo02632h 33506827

[129] Tang WH Wang Z Fan Y Levison B Hazen JE Donahue LM. Prognostic value of elevated levels of intestinal microbe-generated metabolite trimethylamine-N-oxide in patients with heart failure: refining the gut hypothesis. J Am Coll Cardiol (2014) 64(18):1908–14. doi: 10.1016/j.jacc.2014.02.617 PMC4254529 25444145

[130] Shan Z Sun T Huang H Chen S Chen L Luo C. Association between microbiota-dependent metabolite trimethylamine-N-oxide and type 2 diabetes. Am J Clin Nutr (2017) 106(3):888–94. doi: 10.3945/ajcn.117.157107 28724646

[131] Lever M George PM Slow S Bellamy D Young JM Ho M. Betaine and trimethylamine-N-oxide as predictors of cardiovascular outcomes show different patterns in diabetes mellitus: an observational study. PloS One (2014) 9(12):e114969. doi: 10.1371/journal.pone.0114969 25493436 PMC4262445

[132] Shih DM Wang Z Lee R Meng Y Che N Charugundla S. Flavin-containing monooxygenase 3 exerts broad effects on glucose and lipid metabolism and atherosclerosis. J Lipid Res (2015) 56:22–37. doi: 10.1194/jlr.M051680 25378658 PMC4274068

[133] Tang WH Wang Z Levison BS Koeth RA Britt EB Fu X. Intestinal microbial metabolism of phosphatidylcholine and cardiovascular risk. N Engl J Med (2013) 368:1575–84. doi: 10.1056/NEJMoa1109400 PMC3701945 23614584

[134] Papandreou C Bullo M Zheng Y Ruiz-Canela M Yu E Guasch-Ferre M. Plasma trimethylamine-N-oxide and related metabolites are associated with type 2 diabetes risk in the Prevencion con Dieta Mediterranea (PREDIMED) trial. Am J Clin Nutr (2018) 108(1):163–73. doi: 10.1093/ajcn/nqy058 PMC6862602 29982310

[135] Roy S Yuzefpolskaya M Nandakumar R Colombo P Demme RT. Plasma Trimethylamine-N-oxide and impaired glucose regulation: Results from The Oral Infections, Glucose Intolerance and Insulin Resistance Study (ORIGINS). PloS One (2020) 15(1):e0227482. doi: 10.1371/journal.pone.0227482 31940332 PMC6961885

[136] Chakarova N Dimova R Grozeva G Tankova T. Assessment of glucose variability in subjects with prediabetes. Diabetes Res Clin Pract (2019) 151:56–64. doi: 10.1016/j.diabres.2019.03.038 30935927

[137] Mihuta MS Paul C Borlea A Roi CM Velea-Barta OA Mozos I. Unveiling the silent danger of childhood obesity: non-invasive biomarkers such as carotid intima-media thickness, arterial stiffness surrogate markers, and blood pressure are useful in detecting early vascular alterations in obese children. Biomedicines (2023) 11(7):1841. doi: 10.3390/biomedicines11071841 37509481 PMC10376407

[138] O'Donovan G Stensel D Hamer M Stamatakis E. The association between leisure-time physical activity, low HDL-cholesterol and mortality in a pooled analysis of nine population-based cohorts. Eur J Epidemiol (2017) 32:559–66. doi: 10.1007/s10654-017-0280-9 PMC5570782 28667447

[139] Nicholls SJ Lundman P Harmer JA Cutri B Griffiths KA Rye KA. Consumption of saturated fat impairs the anti-inflammatory properties of high-density lipoproteins and endothelial function. J Am Coll Cardiol (2006) 48:715–20. doi: 10.1016/j.jacc.2006.04.080 16904539

[140] Schoeler M Caesar R. Dietary lipids, gut microbiota and lipid metabolism. Rev Endocr Metab Disord (2019) 20(4):461–72. doi: 10.1007/s11154-019-09512-0 PMC6938793 31707624

[141] Fu J Bonder MJ Cenit MC Tigchelaar EF Maatman A Dekens JA. The gut microbiome contributes to a substantial proportion of the variation in blood lipids. Circ Res (2015) 117:817–24. doi: 10.1161/CIRCRESAHA.115.306807 PMC4596485 26358192

[142] Le Chatelier E Nielsen T Qin J Prifti E Hildebrand F Falony G. Richness of human gut microbiome correlates with metabolic markers. Nature (2013) 500:541–6. doi: 10.1038/nature12506 23985870

[143] Koeth RA Wang Z Levison BS Buffa JA Org E Sheehy BT. Intestinal microbiota metabolism of L-carnitine, a nutrient in red meat, promotes atherosclerosis. Nat Med (2013) 19:576–85. doi: 10.1038/nm.3145 PMC3650111 23563705

[144] Koeth RA Levison BS Culley MK Buffa JA Wang Z Gregory JC. γ-Butyrobetaine is a proatherogenic intermediate in gut microbial metabolism of L-carnitine to TMAO. Cell Metab (2014) 20:799–812. doi: 10.1016/j.cmet.2014.10.006 25440057 PMC4255476

[145] Ding L Chang M Guo Y Zhang L Xue C Yanagita T. Trimethylamine-N-oxide (TMAO)-induced atherosclerosis is associated with bile acid metabolism. Lipids Health Dis (2018) 17:286. doi: 10.1186/s12944-018-0939-6 30567573 PMC6300890

[146] Lever M George PM Atkinson W Molyneux SL Elmslie JL Slow S. Plasma lipids and betaine are related in an acute coronary syndrome cohort. PloS One (2011) 6:e21666. doi: 10.1371/journal.pone.0021666 21747945 PMC3128609

[147] Peterson CA Belenchia AM. Vitamin D deficiency & childhood obesity: A tale of two epidemics. Mo Med (2014) 111:49–53. 24645299 PMC6179511

[148] Rajakumar K Moore CG Khalid AT Vallejo AN Virji MA Holick MF. Effect of vitamin D3 supplementation on vascular and metabolic health of vitamin D-deficient overweight and obese children: A randomized clinical trial. Am J Clin Nutr (2020) 111:757–68. doi: 10.1093/ajcn/nqz340 PMC7138671 31950134

[149] Eyupoglu ND Caliskan Guzelce E Acikgoz A Uyanik E Bjørndal B Berge RK. Circulating gut microbiota metabolite trimethylamine N-oxide and oral contraceptive use in polycystic ovary syndrome. Clin Endocrinol (Oxf) (2019) 91:810–5. doi: 10.1111/cen.14101 31556132

[150] Huang J Liu L Chen C Gao Y. PCOS without hyperandrogenism is associated with higher plasma trimethylamine N-oxide levels. BMC Endocr Disord (2020) 20:3. doi: 10.1186/s12902-019-0486-9 31906930 PMC6945624

[151] Eyupoglu ND Ergunay K Acikgoz A Akyon Y Yilmaz E Yildiz BO. Gut microbiota and oral contraceptive use in overweight and obese patients with polycystic ovary syndrome. J Clin Endocrinol Metab (2020) 105:dgaa600. doi: 10.1210/clinem/dgaa600 32860695

[152] Wang Z Roberts AB Buffa JA Levison BS Zhu W Org E. Non-lethal inhibition of gut microbial trimethylamine production for the treatment of atherosclerosis. Cell (2015) 163:1585–95. doi: 10.1016/j.cell.2015.11.055 PMC4871610 26687352

[153] Annunziata G Ciampaglia R Capò X Guerra F Sureda A Tenore GC. Polycystic ovary syndrome and cardiovascular risk. Could trimethylamine N-oxide (TMAO) be a major player? A potential upgrade forward in the DOGMA theory. BioMed Pharmacother (2021) 143:112171. doi: 10.1016/j.biopha.2021.112171 34536755

[154] Jabbour R Ott J Eppel W Frigo P. Carotid intima-media thickness in polycystic ovary syndrome and its association with hormone and lipid profiles. PloS One (2020) 15:e0232299. doi: 10.1371/journal.pone.0232299 32330202 PMC7182264

[155] Patel SS Truong U King M Ferland A Moreau KL Dorosz J. Obese adolescents with polycystic ovarian syndrome have elevated cardiovascular disease risk markers. Vasc Med (2017) 22:85–95. doi: 10.1177/1358863X16682107 28095749 PMC5901904

[156] Cree-Green M Truong U Dorosz JL Moreau K Coe G Baumgartner A. Greater arterial stiffness in polycystic ovary syndrome (PCOS) is an obesity–but not a PCOS-associated phenomenon. J Clin Endocrinol Metab (2010) 95:4566–75. doi: 10.1210/jc.2010-0868 20660051

[157] Maffei S Citti I Guiducci L. Microbiome, sex hormones and cardiovascular risk: a contribution to gender difference. Ital J Gender-Specific Med (2021) 7(1):22–33. doi: 10.1723/3528.35163

[158] Falls JG Ryu D-Y Cao Y Levi PE Hodgson E. Regulation of mouse liver flavin-containing monooxygenases 1 and 3 by sex steroids. Arch Biochem Biophys (1997) 342:212–23. doi: 10.1006/abbi.1997.9965 9186481

[159] Bordoni L Fedeli D Piangerelli M Pelikant-Malecka I Radulska A Samulak JJ. Gender-related differences in trimethylamine and oxidative blood biomarkers in cardiovascular disease patients. Biomedicines (2020) 8(8):238. doi: 10.3390/biomedicines8080238 32717906 PMC7460342

[160] Kander MC Cui Y Liu Z. Gender difference in oxidative stress: A new look at the mechanisms for cardiovascular diseases. J Cell Mol Med (2016) 21:1024–32. doi: 10.1111/jcmm.13038 PMC5387169 27957792

[161] Enck P Zimmermann K Rusch K Schwiertz A Klosterhalfen S Frick JS. The effects of maturation on the colonic microflora in infancy and childhood. Gastroenterol Res Pract (2009) 2009:752401. doi: 10.1155/2009/752401 19763278 PMC2744901

[162] Agans R Rigsbee L Kenche H Michail S Khamis HJ Paliy O. Distal gut microbiota of adolescent children is different from that of adults. FEMS Microbiol Ecol (2011) 77(2):404–12. doi: 10.1111/j.1574-6941.2011.01120.x PMC4502954 21539582

[163] Yuan X Chen R Zhang Y Lin X Yang X. Gut microbiota: effect of pubertal status. BMC Microbiol (2020) 20:334. doi: 10.1186/s12866-020-02021-0 33143658 PMC7640488

