# Supplementary material for: Connections between serum Trimethylamine N-Oxide (TMAO), a gut-derived metabolite, and vascular biomarkers evaluating arterial stiffness and subclinical atherosclerosis in children with obesity
Source: Front Endocrinol (Lausanne). 2023 Oct 2;14:1253584. doi: 10.3389/fendo.2023.1253584 (PMC10577381; doi:10.3389/fendo.2023.1253584)
Supplement: Supplementary file 1 [file DataSheet_1.pdf]

Supplementary Material

Connections between Serum Trimethylamine N-Oxide (TMAO), a Gut-Derived Metabolite, and Vascular Biomarkers Evaluating Arterial Stiffness and Subclinical Atherosclerosis in Children with Obesity

Supplementary Figures

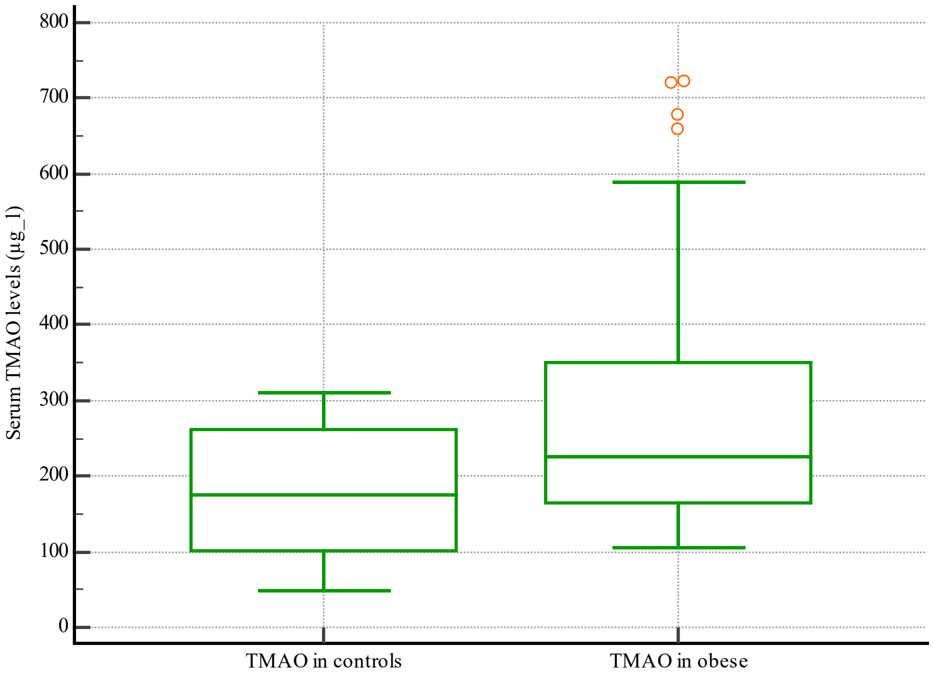

Supplementary Figure 1. Comparison of median serum TMAO levels between obese subjects and controls (p=0.02).

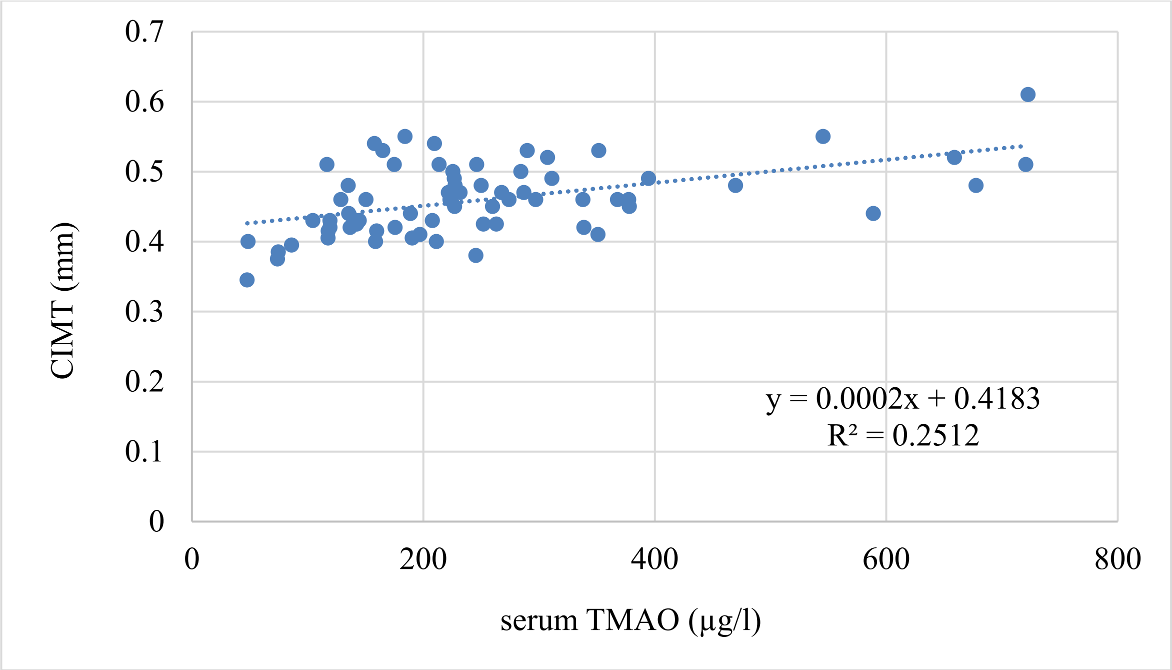

Supplementary Figure 2. Correlation between serum TMAO and CIMT values in all participants (ρ=0.48, *p*=<0.001)

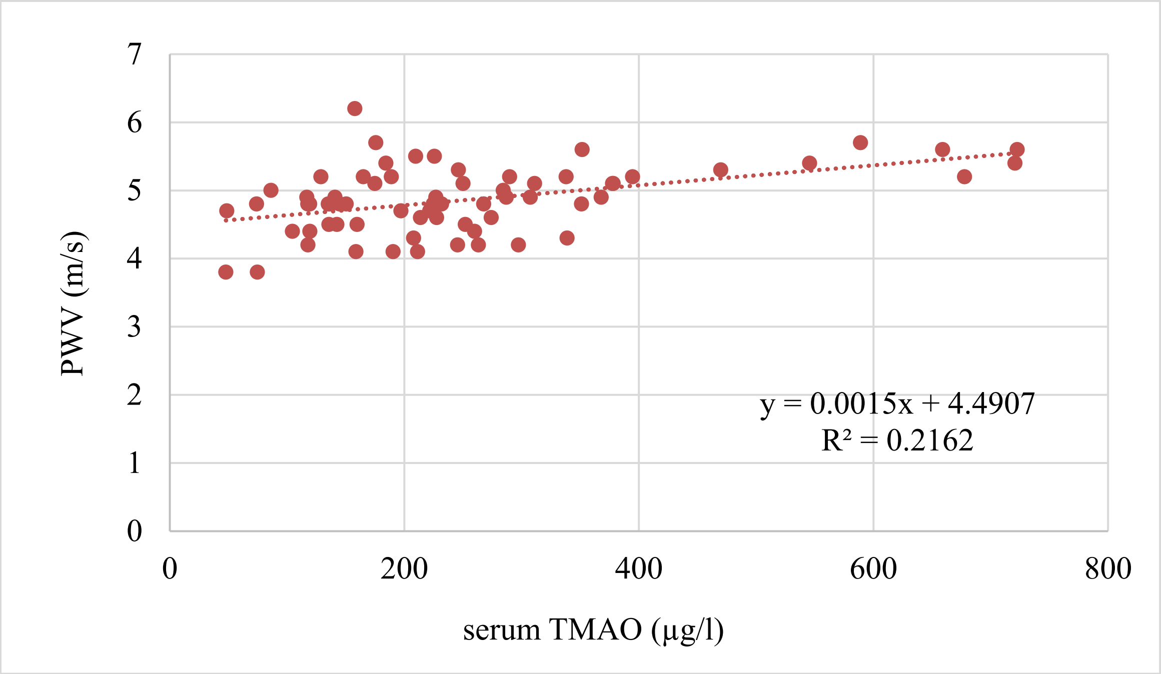

Supplementary Figure 3. Correlation between serum TMAO and PWV values in all participants (ρ=0.41, *p*=0.0004)

Supplementary Table 1. Differences between median TMAO values between sexes, for all participants (Mann-Whitney tests).

	Serum TMAO median values		
Sex	Obese	Controls	*p*
Male	224	261.6	0.62
Female	287	114.45	**0.0006**
p	0.32	**0.002**	

*The significant results in this table were bolded.

Supplementary Table 2. Differences between median TMAO values between age groups, for all participants (Krussal-Wallis H-test, Dunn-Bonferroni-Tests for the two-by-two comparisons).

	Median values				*p*		
	<12 y	12-15 y	≥15 y	H-test *p*	< 12 y vs. 12-15 y	<12 y vs. ≥15 y	12-15 y vs. ≥ 15 y
Serum TMAO	226.9	217.3	226.6	0.37	0.28	0.7	0.19

Supplementary Table 3. Differences between median TMAO values between stage development groups, for all participants (Krussal-Wallis H-test, Dunn-Bonferroni-Tests for the two-by-two comparisons).

	Median values				*p*		
	Tanner 1	Tanner 2-4	Tanner 5	H-test *p*	Tanner 1 vs Tanner 2-4	Tanner 1 vs. Tanner 5	Tanner 2-4 vs. Tanner 5
Serum TMAO	245.5	221.8	225.7	0.59	0.31	0.68	0.56

Supplementary Table 4. One-way ANOVA posthoc tests comparing data regarding obese girls' menstrual cycles (the two-by-two comparisons present Bonferroni-corrected p-values).

	Mean values				*p*		
	Without menarche	Regular menses	Irregular menses	ANOVA *p*	without menarche vs. regular menses	without menarche vs. irregular menses	regular menses vs irregular menses
Serum TMAO	353.625	171.27	309.08	0.15	0.24	0.98	0.18
BMI	22.44	35.09	34.58	0.07	0.18	0.15	1
WC	93.75	106.75	105.14	0.09	0.24	0.12	1
WHR	0.67	0.64	0.65	0.9	1	1	1
HOMA-IR	3.73	2.8	3.15	0.69	1	1	1

**p*=1 for the two-by-two comparisons represents the maximum p-value after the Bonferroni correction.
